# Instructing cells with programmable peptide DNA hybrids

**DOI:** 10.1038/ncomms15982

**Published:** 2017-07-10

**Authors:** Ronit Freeman, Nicholas Stephanopoulos, Zaida Álvarez, Jacob A Lewis, Shantanu Sur, Chris M Serrano, Job Boekhoven, Sungsoo S. Lee, Samuel I. Stupp

**Affiliations:** 1 Simpson Querrey Institute for BioNanotechnology, Northwestern University, 303 East Superior Street, Chicago, Illinois 60611, USA; 2 Department of Biomedical Engineering, Northwestern University, 2145 Sheridan Road, Evanston, Illinois 60208, USA; 3 Department of Materials Science and Engineering, Northwestern University, 2220 Campus Drive, Evanston, Illinois 60208, USA; 4 Department of Chemistry, Northwestern University, 2145 Sheridan Road, Evanston, Illinois 60208, USA; 5 Department of Medicine, Northwestern University, 251 East Huron Street, Chicago, Illinois 60611, USA

## Abstract

The native extracellular matrix is a space in which signals can be displayed dynamically and reversibly, positioned with nanoscale precision, and combined synergistically to control cell function. Here we describe a molecular system that can be programmed to control these three characteristics. In this approach we immobilize peptide-DNA (P-DNA) molecules on a surface through complementary DNA tethers directing cells to adhere and spread reversibly over multiple cycles. The DNA can also serve as a molecular ruler to control the distance-dependent synergy between two peptides. Finally, we use two orthogonal DNA handles to regulate two different bioactive signals, with the ability to independently up- or downregulate each over time. This enabled us to discover that neural stem cells, derived from the murine spinal cord and organized as neurospheres, can be triggered to migrate out in response to an exogenous signal but then regroup into a neurosphere as the signal is removed.

## Abstract (web summary)

The extracellular matrix can affect cell behaviour both physically and biochemically. Here, the authors developed a substrate that is based on peptides and nucleic acids hybrids that can dynamically present signals upon demand which regulate cell adhesion and migration, thereby controlling cell organisation.

The environment of a cell is heterogeneous and dynamic, providing both structural and biochemical support. In particular, the extracellular matrix (ECM) controls the dynamics, spatial positioning, and combinatorial synergies of signals in order to direct cell behaviour^1^,^2^. Artificial forms of this matrix for tissue regeneration need to recapitulate these three characteristics in a single system. Most efforts in this area have effectively addressed only one of these three key phenomena, and focused mainly on static cell adhesion^3^ or irreversible switching of bioactivity^4^,^5^,^6^,^7^. Also, all of these systems have been designed to respond to one stimulus and promote one predefined cellular response. To better mimic the multi-responsive nature of the native ECM it is therefore necessary to design materials that are able to respond to multiple signals orthogonally and reversibly. DNA is a good candidate for anchoring bioactive signals to a substrate and mediating their interactions with cells due to its highly programmable nature, as highlighted in three recent reports that used Watson–Crick pairing as a molecular tension probe to measure integrin forces during cell adhesion^8^,^9^,^10^. Previous work also utilized DNA hybridization to immobilize cells on surfaces^11^,^12^,^13^, and as an interesting approach to cluster cells *in vivo*^14^.

Here we describe the use of DNA to dynamically control and spatially arrange multiple bioactive signals in order to mimic the features of the extracellular matrix in a single system. In this approach, a peptide-DNA (P-DNA) molecule is immobilized on a surface through complementary DNA tethers. By engineering a series of tethers responsive to different stimuli, we show that cells can adhere and spread on the surface reversibly. The use of P-DNA in cell signalling allows multiple cycles of reversibility by simply adding soluble biologically compatible molecules such as DNA and enzymes, without the need for external stimuli like photons or electrochemical potentials. The DNA can also be used as a molecular ruler to control the spacing between two adhesion peptides, or to tether both growth factors and bioactive peptides simultaneously in order to explore synergistic effects. Finally, by designing orthogonal DNA handles we were able to selectively and reversibly present two different peptide signals (a differentiation signal for neural stem cells and a growth factor-mimetic signal for their proliferation) with the ability to independently up- or downregulate each over time. This enabled us to discover that neural stem cells organized as neurospheres derived from the postnatal murine spinal cord can be triggered to migrate out driven by the differentiation signal and then regroup as the signal is switched off. This phenomenon was observed over multiple cycles. This reveals for the first time that strong cell–cell interactions persist in neurospheres to a degree that can reverse their migration. Furthermore, with this capability in hand, one can follow not only the phenotype of cells during these dynamic processes, but also their accompanying re-organization within the neurosphere. This platform presents an attractive model to study the important role cell–matrix interactions play within the stem cell niche and provides new avenues for developing rationally designed dynamic regenerative biomaterials.

## Results

### Synthesis and bioactivity of peptide DNA modified surfaces

**Figure 1:**
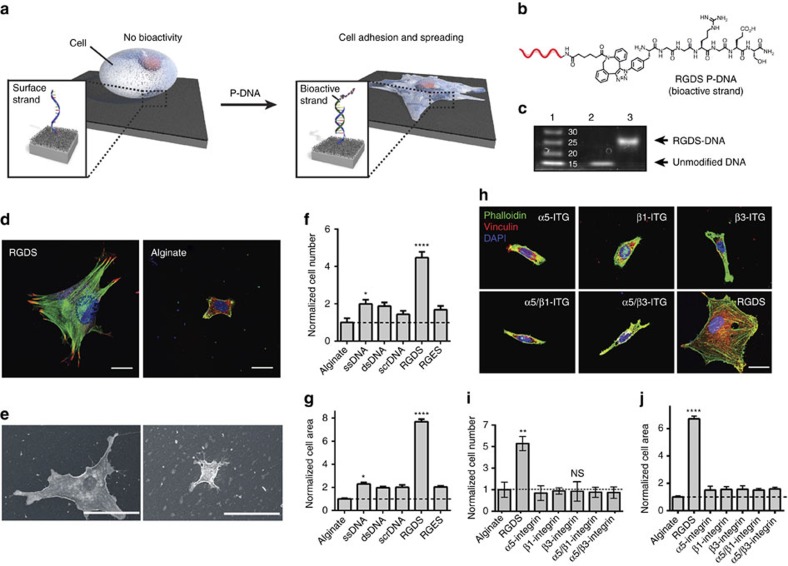
Cell adhesion on RGDS P-DNA modified surfaces. (**a**) Schematic representation of fibroblast adhesion and spreading. (**b**) Chemical structure of RGDS P-DNA (DNA strand is represented schematically in red). (**c**) Polyacrylamide gel electrophoresis of RGDS P-DNA: lane 1, molecular weight ladder, lane 2, unmodified DNA, lane 3, purified DNA-RGDS conjugate. (**d**) Confocal microscopy images of fibroblasts 24 h after being plated on either RGDS P-DNA or alginate surfaces, (**e**) scanning electron micrographs of fibroblasts 24 h after being plated on either RGDS P-DNA or alginate surfaces . Scale bars for fluorescent images; 20 μm and for micrographs: 50 μm. (**f**) Normalized cell number and area (**g**) of fibroblasts plated on various coated surfaces (all values were normalized relative to alginate, *n*=5). (**h**) Confocal images of fibroblasts treated with integrin-blocking antibodies and plated on RGDS P-DNA surfaces for 24 h (staining for actin (phalloidin, green), vinculin (red) and nuclei (DAPI, blue) reveals the cytoskeletal organization, focal adhesions and cell nuclei, respectively. Scale bar: 20 μm. (**i**) Normalized number and area (**j**) of cells from part **h**. All values were normalized relative to alginate; *n*=3. (*P<0.05; **P<0.01; ****P<0.0001).

In the P-DNA platform, a peptide or protein is covalently linked to a DNA tether, resulting in a conjugate we term the ‘bioactive strand’. This strand is then immobilized on a solid surface through a complementary DNA, which we term the ‘surface strand’ (Fig. 1a). Cells are plated on the modified surface and their membrane receptors interact with the bioactive strand to influence cell behaviour. To promote cell adhesion, we attached the peptide RGDS^15^ (derived from fibronectin) to ssDNA using copper-free click chemistry^16^ (for a list of all DNA sequences used, see Supplementary Table 1). The resulting RGDS-DNA conjugate (Fig. 1b) was purified using reverse-phase HPLC and both polyacrylamide gel electrophoresis and ESI-MS analyses confirmed its identity and high purity (Fig. 1c; Supplementary Fig. 1). The RGDS P-DNA was then hybridized to the complementary surface strand covalently linked to an alginate-coated glass surface^7^. We selected alginate due to its biocompatibility and its ability to resist cell adhesion while being easily modified with amine-functionalized DNA strands (see Supplementary Figs 2 and 3 for synthesis and characterization of surfaces).

We used fibroblast cell adhesion and spreading as a functional assay for RGDS-mediated integrin activation^17^. Substrates modified with the surface DNA strand were treated with either RGDS-DNA or P-DNA containing the non-bioactive peptide RGES^4^. After 1 h, unbound P-DNA was removed by rinsing and mouse fibroblast cells were plated on the modified substrates and allowed to spread for various times, after which the number of bound cells and their surface area were quantified (for the effect of RGDS density, the availability of the epitope and the analysis of cell area over time see Supplementary Fig. 4). Differences in cell attachment to RGDS surfaces in comparison with unmodified alginate were detected as early as 30 min after the cells were seeded on the substrates (Supplementary Fig. 4). After 24 h, we observed roughly four times as many cells adhered to RGDS surfaces relative to unmodified alginate (alginate) or to surfaces modified with single stranded DNA (ssDNA) or double stranded DNA (dsDNA) lacking any peptide (Fig. 1f). Surfaces modified with surface DNA and treated with RGDS conjugated to a scrambled DNA sequence (scrDNA) resulted in poor cellular adhesion, confirming that the peptide was bound to the surface by DNA hybridization (Fig. 1f). At the same time, fibroblasts plated on RGES showed similar cell numbers relative to alginate, validating the specificity of the bioactive peptide. We also investigated the extent of cell spreading on the different surfaces (Fig. 1g). We found that cells plated on RGDS surfaces showed a sixfold increase in cell area after 24 h relative to the unmodified substrate. Both confocal and scanning electron microscopy images show cells with a polygonal and highly spread morphology, with well-defined actin stress fibers and focal adhesions (Fig. 1d; Supplementary Fig. 4). By contrast, cells plated on RGES did not demonstrate any increase in cell area, again confirming the bioactive role of the peptide (Fig. 1g). To determine whether cell binding and spreading was mediated by RGDS–integrin activation, we used antibodies to block the relevant integrins (α_5_β_1_, α_v_β_3_). Under these conditions we observed poor attachment of cells and no spreading, indicating that bioactivity induced by the RGDS surfaces relies upon integrin engagement (Fig. 1h–j).

### Reversible control of bioactivity

**Figure 2:**
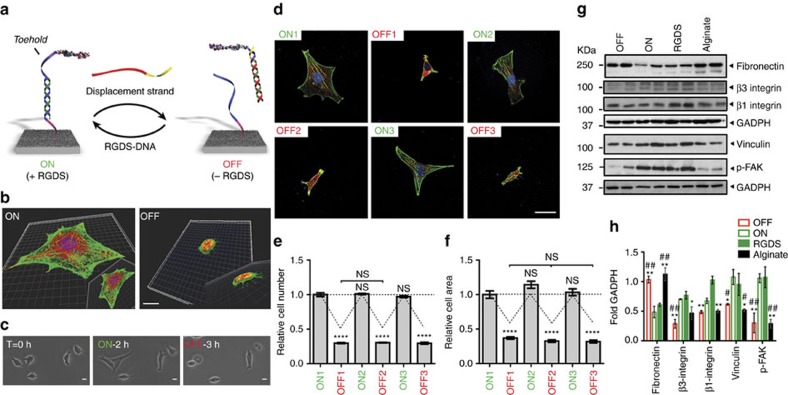
Dynamic reversible switching of cell bioactivity. (**a**) Schematic representation of toehold-mediated strand displacement system. Engineering a toehold into the bioactive strand allows its removal by a fully complementary displacement strand, thus switching bioactivity OFF; this process regenerates the surface strand, allowing for a second addition of the RGDS-DNA to turn bioactivity back ON. This process can be repeated for multiple cycles. (**b**) Three dimensional shadow projection image of confocal sections of fibroblasts on surfaces where RGDS was displayed (ON state after 2 h), and then removed using strand displacement (OFF state after 3 h). (**c**) Representative time lapse images of fibroblasts on surfaces in the ON and OFF states (phase contrast). Scale bar: 10 μm. (**d**) Confocal micrographs of cells on surfaces where the bioactivity has been switched ON and OFF for multiple cycles using the toehold mechanism . Staining for actin (phalloidin, green), vinculin (red) and nuclei (DAPI, blue) reveals the cytoskeletal organization, focal adhesions and cell nuclei, respectively. Scale bar: 20 μm (**e**) Normalized cell number and area (**f**) (relative to unmodified alginate; *n*=3) for each of the ON–OFF cycles shown in **d**. (**g**) Western blot analysis of protein expression (related to matrix deposition, integrin activation and stability of focal adhesions) in fibroblasts measured at various ON and OFF states. (**h**) Relative expression of proteins derived from western blots in **g**. All values were normalized to GAPDH expression, triplicate samples were analysed. (*P<0.05; **P<0.01; ****P<0.0001; ^#^*P*<0.05; ^##^*P*<0.01; * relative to ON1, ^#^ relative to ON2).

We next investigated the reversible switching of bioactivity using the unique properties of DNA to emulate the dynamic nature of ECMs in biological systems. Previous studies have used strategies like enzymatic cleavage^5^, thermoresponsive polymers^18^, electroswitching^19^, photocleavage^4^,^20^,^21^,^22^, photoisomerization^20^ or host-guest displacement^6^,^7^ to dynamically control cell adhesion. Most of these approaches, however, are limited to one bioactive signal and rarely offer reversibility over multiple cycles. In contrast, using DNA as a functional tether allowed us to present and remove the bioactive signal in a programmable and highly reversible manner. To control the bioactivity of the peptide we introduced a short single-stranded sequence (referred to as a ‘toehold’) into the bioactive strand. Adding a fully complementary DNA trigger to the bioactive strand enables its removal from the surface using a mild process and in programmable manner, while regenerating the surface strand for binding a second P-DNA molecule (Fig. 2a). We define the ON state as one where the RGDS epitope is functional, resulting in cell spreading, and the OFF state where the signal is removed or is otherwise inaccessible, which should inhibit spreading (Fig. 2b). We first analysed the kinetic response of the OFF state upon addition of the DNA stimulus (Fig. 2c). In the initial ON state cells were allowed to spread for 2 h (Fig. 2d,e), and after addition of the displacement strand the cells reverted back to the area of cells on the unmodified surfaces (and some of the cells detached from the surface). The strand displacement required to transition between the ON and the OFF states is extremely fast (in the order of a few minutes) compared to the time it takes cells to react to the change of state (Supplementary Figs 6g and 7f). This dynamic behaviour was also followed by live cell time-lapse videos showing the dynamic response of the same cells to the presentation and subsequent removal of the RGDS signal (Supplementary Movies 1–5).

One key advantage of this approach is that switching to the OFF state regenerates the free surface strand, thus allowing the reintroduction of RGDS P-DNA to restore the ON state. Cells that detached or contracted during the OFF state were able to reattach and spread normally on the subsequent ON step. The switching of bioactivity between ON and OFF states was demonstrated over several cycles by simply adding soluble single-stranded DNA to cell cultures, resulting in repeated cell spreading and contraction (Fig. 2d–f). To address whether the tailored surfaces facilitate this observed dynamic behaviour by activating integrin signalling resulting in focal adhesion formation, we followed the expression of several key proteins in cells exposed to the different ON and OFF states (Fig. 2g,h). Cells cultured on surfaces presenting RGDS (ON 1) exhibited higher levels of vinculin and phosphorylated focal adhesion kinase (FAK) relative to cells cultured on alginate or upon removal of the RGDS signal (OFF 1). These results demonstrate that surfaces presenting the RGDS bioactive peptide promote the formation of focal adhesions and stimulate downstream kinase activity associated with integrin signalling. Interestingly, when the RGDS was re-displayed on the surface (ON 2), the levels of FAK phosphorylation and vinculin expression were similar to the ones obtained for cells after the first ON step (ON 1), confirming that the bioactivity can be switched repeatedly between the different states. It is therefore possible through this platform to trigger changes in cell signalling using DNA-driven dynamic display of an exogenous signal. In addition, cells cultured on RGDS surfaces (ON states) exhibited higher levels of β1 and β3 integrins, while expressing lower levels of fibronectin relative to surfaces lacking the bioactive peptide (alginate, OFF states). These results confirm that the bioactivity is indeed mediated by the displayed peptide through integrin activation and not by secondary deposition of ECM proteins secreted by the cells. To further establish the role of fibronectin secretion by the cells we visualized the protein on the various surfaces using confocal optical microscopy (Supplementary Fig. 5). The images show formation of fibronectin fibrillar matrix on the alginate as well as on poly-D-lysine modified surfaces, but none is observed on the RGDS-modified surfaces. These results confirm further that the bioactive RGDS peptide displayed on the surface is mediating the adhesion and spreading of the cells.

In addition to toehold-mediated strand displacement, we explored two other orthogonal DNA-mediated mechanisms for dynamic signal presentation including the reversible switching of the conformation of a DNA hairpin and selective degradation of the bioactive strand using a nuclease enzyme. Both of these systems similarly allowed for reversible switching between the ON and OFF states over multiple cycles, further demonstrating the versatility of the P-DNA platform (Supplementary Fig. 8).

### Promoting distance dependent synergies

**Figure 3:**
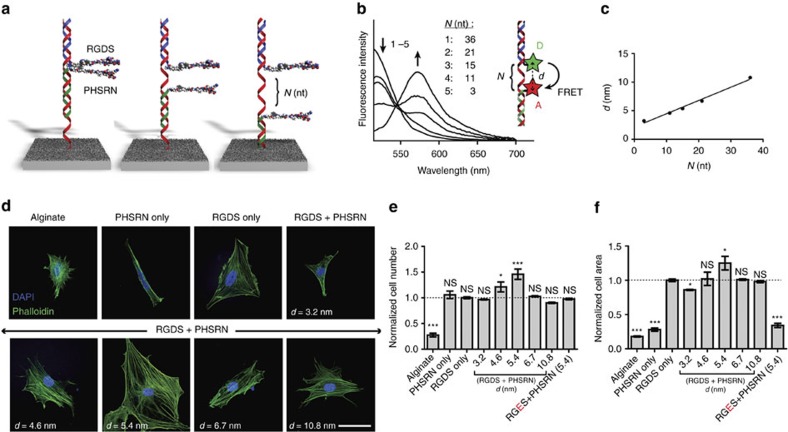
Synergy between RGDS and PHSRN. (**a**) DNA surface strands with variable spacer regions were used to tune the distance between the peptide signals RGDS and PHSRN. (**b**) Fluorescence resonance energy transfer traces for DNA constructs modified with donor (**D**) and acceptor (**A**) dyes to probe the distance between them. (**c**) Relationship between *N* and the distance *d* as determined by FRET. (**d**) Confocal microscopy images of cells on indicated surfaces; staining for actin (phalloidin, green), and nuclei (DAPI, blue) reveals the cytoskeletal organization and cell nuclei, respectively. Scale bar: 50 μm, relevant to all images. (**e**) Normalized cell number and area (**f**) of fibroblasts on the surfaces indicated relative to the RGDS-only value; *n*=3. (*P<0.05; ***P<0.001).

A second challenge in mimicking the ECM is positioning multiple signals with nanoscale precision. For example, the peptides RGDS and PHSRN (derived from the 10th and 9th type III domains of fibronectin, respectively) have been shown to operate synergistically to promote cell adhesion and spreading^23^,^24^. Changing the distance between epitopes on a biomaterial scaffold has suggested that this synergy is spatially dependent^25^. While several strategies to investigate the effect of interligand spacing of RGDS on surfaces were established^26^,^27^,^28^, controlling the nanoscale spacing between two different signals is underexplored. DNA is an ideal scaffold for precisely spacing multiple biological ligands due to its well-understood structural properties^29^,^30^. As described below, the effect of distance between RGDS and PHSRN on their synergy was probed using a series of surface strands that systematically tune the separation (*N*, in nucleotides) between P-DNA strands bearing these epitopes (Fig. 3a; Supplementary Fig. 9). We varied *N* to span both larger and smaller distances than the native separation of the signals in fibronectin (∼5 nm), and calculated the distance (*d*, in nm) using FRET with a donor and acceptor pair in place of RGDS and PHSRN, respectively (Fig. 3b,c). We observed the greatest distance-dependent synergy between RGDS and PHSRN at a separation of 5.4 nm as indicated by an increase in cell number by 46% and cell area by 25% after 17 h relative to surfaces with RGDS alone (Fig. 3d–f). At both shorter and longer distances this synergy was no longer observed. Also, replacing RGDS with the non-bioactive peptide **RGES** did not promote a synergistic effect, confirming the specific bioactive role of the peptide and demonstrating that both RGDS and PHSRN signals are required. We point out that the 5.4 nm value closely resembles the natural distance between the two peptide epitopes in fibronectin, highlighting the utility of DNA for probing natural distance-dependent synergies in the ECM. More specifically, this experiment demonstrates how this capability could be used to optimize signal display by artificial scaffolds or surfaces.

A third important role of the native extracellular matrix is to bind growth factors, increasing their local concentration and enhancing their activity by co-localization of their corresponding receptors with integrins^2^,^31^. Our P-DNA platform enables the control of this process by using two orthogonal surface DNA strands to bind the integrin ligand and a growth factor of interest. We chose to investigate the synergy between RGDS epitopes and bone morphogenetic protein (BMP-2), a therapeutically relevant growth factor for inducing osteogenesis^32^. Interestingly, when we plated C2C12 premyoblast cells on surfaces with both RGDS and BMP2 we saw a significant increase in differentiated osteoblasts cells relative to surfaces modified with just BMP2 or RGDS, demonstrating that the synergy of the two signals leads to a marked enhancement of growth factor activity. (Supplementary Figs 10 and 11).

### Orthogonal reversible display of multiple biomimetic signals

**Figure 4:**
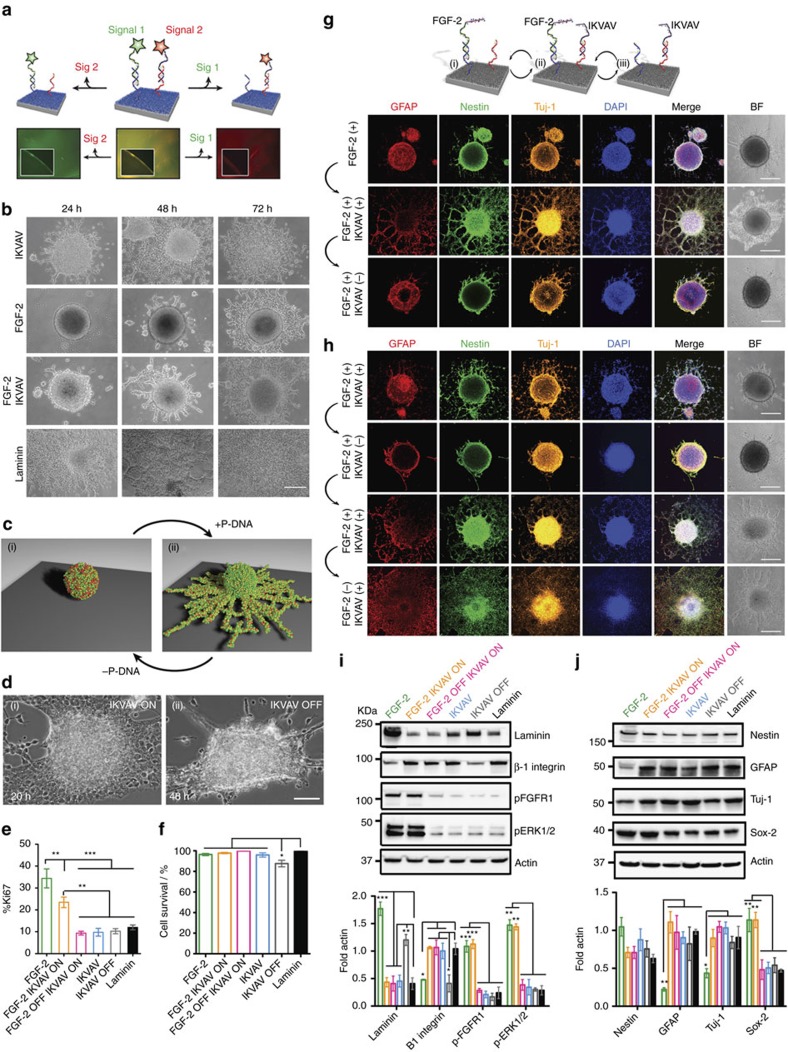
Dynamic control over multiple signals and its effect on neural stem cells. (**a**) Surfaces modified with both red and green fluorescent strands bearing orthogonal toeholds, allowing the selective removal of either signal using its complementary displacement strand. (**b**) Bright-field microscopy images of neural stem cells on surfaces bearing indicated signals at different time points. Scale bar: 50 μm. (**c**) Schematic of reversible migration of neural stem cells out of and back into the neurosphere upon dynamic presentation of a P-DNA signal. (**d**) Representative bright field time lapse images of neural stem cells migrating out of a neurosphere in response to the IKVAV signal (i), and back into the neurosphere (ii) after removal of the signal. Scale bar: 50 μm. (**e**) Percent of ki67 positive proliferative cells and cell survival (**f**) on indicated surfaces. (**g**,**h**) Immunofluorescence images of neurospheres subjected to dynamic and orthogonal presentation of IKVAV and FGF-2 signals. Scale bar: 50 μm, and relevant to all images. (**i**,**j**) Western blots and normalized protein levels of neural stem cells on modified surfaces. All values were normalized to actin expression; triplicate samples were analysed (*P<0.05; **P<0.01; ***P<0.001).

We next explored use of the P-DNA platform to enable dynamic display of multiple signals using bioactive strands responsive to orthogonal triggers. As a proof of principle we first constructed substrates modified with two different surface strands, one to attach the green fluorophore fluorescein and another one to bind the red fluorophore TAMRA using their respective complementary strands. The simultaneous display of both fluorophores (mimicking two bioactive epitopes) was evidenced by the yellow fluorescence of the surfaces (Fig. 4a). Since the fluorophore-modified strands were designed to be responsive to orthogonal toehold sequences, we were able to selectively generate green or red fluorescence on the surfaces. This dynamic capability applied to bioactive epitopes could be used to investigate in a programmable manner how cells respond to temporal variations in their environment. We were specifically interested in exploring how artificial matrices mediate the proliferation and differentiation of neural stem cells (NSCs) and how these two processes could be temporally controlled with the P-DNA platform. NSCs can be maintained *in vitro* under certain culture conditions as clusters of cells termed neurospheres. The population of cells that constitute a neurosphere is multipotent, capable of generating multiple cell types (neurons, astrocytes and oligodendrocytes)^33^. Subjecting neurospheres to different environmental cues using the P-DNA platform offers an attractive model to study and uncover the effect of varying molecular conditions on neural stem cell behaviour and can provide insights into ECM-cell interactions, which are thought to play an important role within the neural stem cell niche^33^,^34^,^35^,^36^,^37^,^38^. We therefore applied our system to neurospheres comprised of NSCs isolated from the postnatal murine spinal cord which are potentially interesting from a translational point of view. Surfaces modified with two orthogonal DNA strands were used to independently and reversibly control the presentation of two peptide epitopes. One epitope could activate neural stem cell differentiation, and a second one could stimulate their proliferation (for the synthesis of the peptide-DNA bioactive signals see Supplementary Fig. 12). We chose the short peptide signal IKVAV^39^ found in Laminin-1, which was previously demonstrated to mediate neuronal differentiation^40^, and as the proliferative signal we selected a peptide known to be a mimic of the mitotic growth factor fibroblast growth factor 2 (refs 41, 42, 43; FGF-2). Sequential proliferation followed by migration and differentiation of neural stem cells can prove interesting for transplanted stem cells in a biomaterial to generate more cells and then allow them to migrate outwards to repopulate a lesion, for example in the case of spinal cord or brain injuries.

First, we explored the effect of the persistent display of either one bioactive signal or both signals on neurospheres and followed their attachment, migration, proliferation, differentiation and organization over time (Supplementary Figs 13 and 14). When the IKVAV signal was displayed, cells were driven to migrate out from the neurosphere, generating a flatter structure similar to that on laminin-coated surfaces (Fig. 4b,e; Supplementary Movies 6, 7 and 14). We quantified migration as the total area of neurosphere normalized to the largest inner circle. The cellular migration from the neurosphere as well as neuronal differentiation is in agreement with previous reports investigating NSC response when exposed to laminin-1 (refs 34, 35, 36) or IKVAV-containing materials^40^,^44^,^45^. Presentation of the FGF-2 mimic peptide signal promoted proliferation of the cells, while maintaining the spherical nature of the aggregate (Supplementary Figs 13, 14 and 18a). This is evident by the larger sizes of the neurospheres as well as the increased number of cells expressing ki67, a marker for proliferating cells^46^ (Fig. 4b; Supplementary Figs 13 and 18a,b). At later time points (after 72 h) cell migration was also observed in these neurospheres (Fig. 4b; Supplementary Fig. 14). By presenting both IKVAV and FGF-2 peptide signals we observed migration as well as proliferation (Fig. 4b,e). We also confirmed that cells remained viable for at least 3 days when they were cultured on the different bioactive surfaces (Fig. 4f; Supplementary Fig. 18c,d). In addition, we found that the effects shown were specifically triggered by the bioactive peptide signals. Substrates modified with surface DNA and treated with scrambled peptide sequences (scrIKVAV; scrFGF-2) resulted in poor cellular adhesion, similarly to cells plated on alginate, validating the specificity of the bioactive peptides (Supplementary Figs 15 and 16). When neurospheres were plated on surfaces modified with two different peptides, a bioactive peptide signal and a scrambled one (IKVAV and scrFGF-2 or FGF-2 and scrIKVAV), the cells showed similar effects to the ones observed on surfaces modified with solely the bioactive peptide, further confirming the bioactive role of the peptide signal (Supplementary Fig. 16). We next examined whether the IKVAV- and FGF-2- modified surfaces promote cell migration and proliferation through the β1-integrin and FGF receptor 1, respectively by following their expression by western blot analysis after 24 h (Fig. 4i). β1-integrin was highly expressed in cells cultured on IKVAV-modified surfaces as well as on laminin, while FGF receptor 1 was expressed only in cells plated on surfaces presenting the FGF-2 peptide. Furthermore, when the FGF-2 peptide was displayed cells exhibited a higher expression of the phosphorylated extracellular signal regulated kinase (ERK1/2), which is a downstream marker for the FGF-2 signalling pathway. We also measured laminin expression in the various conditions in order to determine the effect of ECM proteins secreted by the cells on neural cell behaviour. Low levels of laminin were detected when IKVAV signal was displayed, while high expression of laminin was observed for the surfaces presenting solely the FGF-2 peptide signal (Fig. 4i). Taken together, these results demonstrate that by altering the exogenous environment of NSCs, expressions of the ECM protein laminin and of β1-integrin within the neurosphere are modified. We also observed (even after a short time point, 24 h) differences in the phenotypic profiles as well as their distributions throughout the neurospheres when comparing cells plated on IKVAV versus those exposed to the FGF-2 peptide. The IKVAV signal triggered higher expression of differentiated neuronal (Tuj-1 positive) and astroglial (GFAP positive) cell markers whereas expression of the neural stem cell marker sox-2 was reduced. On the other hand, FGF-2 promoted the opposite response, resulting in augmented production of immature cell markers and reduced levels of mature neural cell markers (Fig. 4j).

We utilized next the P-DNA platform to probe the dynamic presentation of the IKVAV and FGF-2 signals on neural cell behaviour (migration, proliferation, differentiation and organization) by displaying and removing each signal at defined time points using DNA-mediated strand displacement. Interestingly, after cells migrated out of the neurosphere driven by IKVAV signal presentation (ON state), we observed a surprising regrouping of the cells into a neurosphere after the signal was removed (OFF state) (Fig. 4c,d; Supplementary Movies 8, 9). This suggests that even after cells have migrated out of the neurosphere, strong cell-cell interactions persist to a degree that can reverse the process. Furthermore, the display and subsequent removal of IKVAV was accompanied by reorganization of cells within the neurosphere (Supplementary Fig. 17d). This change in signal presentation led to differences in the expression levels of β1-integrin as well as laminin (Fig. 4i). When the IKVAV signal was removed, expression of β1 integrin decreased significantly accompanied by elevated production of laminin. The P-DNA platform enabled switching between ON and OFF states over several cycles, as demonstrated by repeated cell migration out and back into the neurosphere, accompanied by cellular reorganization (Fig. 4d; Supplementary Fig. 17d; Supplementary Video 10).

Finally, we utilized surfaces responsive to two orthogonal triggers (Fig. 4g,h) in order to explore the dynamic presentation of the two biological signals on neural cell behaviour. Two different dynamic patterns were investigated, the first involved the sequential presentation of signals (Fig. 4g), and the second included the simultaneous display of both signals followed by the selective removal of each at different time points (Fig. 4h). We found that addition of the IKVAV signal 24 h after exposure of the cells to the FGF-2 peptide triggered their migration out of the neurosphere and induced differentiation (Fig. 4g,j; Supplementary Video 11). Upon removal of the IKVAV signal (after 48 h) the migrating cells retracted and distributed mostly on the outer region of the sphere (Fig. 4g). We envision that this type of sequential signal exposure could enable the implementation of controlled proliferation of stem cells, which can be subsequently activated for selective differentiation upon further exposure to another signal. Also, the potential to regroup differentiated cells by removing the signal can enable further processing of cells.

The second dynamic signalling mode we explored involved the selective display of one of two epitopes after the system was exposed to both stimuli (Fig. 4h). After subjecting cells to both the IKVAV and FGF-2 peptide signals simultaneously, both proliferation and migration of cells out of the neurosphere were observed. Using one of the two DNA orthogonal stimuli, we selectively switched the IKVAV signal OFF and then ON while keeping the FGF-2 peptide signal ON. This signal modulation triggered the reversible migration of cells into and out of the neurosphere, and also altered their spatial distribution in the aggregate (Fig. 4h). We then switched OFF the FGF-2 peptide signal using the second DNA stimulus but maintained the IKVAV signal in the ON state. This triggered migration of cells out of the neurosphere (Fig. 4h; Supplementary Movies 12 and 13). Interestingly, when the FGF-2 peptide signal was turned OFF we observed a decrease in the phosphorylated FGF receptor and phosphorylated ERK1/2 expression (Fig. 4i). This established that manipulation of the exogenous signal triggered changes in cell signalling.

## Discussion

The P-DNA platform was able to induce reversible biological cell adhesion over multiple cycles, optimize at the nanometre scale the distance between two signals in the matrix for synergistic signalling of the cell, and probe the individual roles of two different localized signals in the matrix, a biological adhesion epitope and a growth factor. Furthermore, the P-DNA strategy has enabled experiments in which neurospheres can be subjected to different temporally active signals. This allowed us to discover that once cells are triggered to migrate out of neurospheres, they remain engaged by cell-cell interactions that unexpectedly reverse their migration upon removal of the signal. Identifying the nature of these cell-cell interactions is however beyond the scope of this paper. Furthermore, the capability to dynamically switch signals ON and OFF reversibly allowed us to follow the phenotype of cells during these dynamic processes. This unprecedented observation on a neurosphere was uniquely enabled by our dynamic platform controlling the temporal presentation of the signals and would not have been possible by merely subjecting the cells to a persistent signal. We hypothesize that the strategy described here to control dynamic bioactivity will open opportunities in the manipulation of cells and their phenotypes as signals are displayed reversibly with temporal control. The P-DNA technology is not limited to the display of peptide cues. Since for many biological signals and matrix proteins such mimetic peptides are not available, this approach can also be adopted for the display of proteins by modifying the protein with a DNA tether, as exemplified in Supplementary Figs 10 and 11. Because the synthetic modification of proteins can potentially limit their activity or availability, these steps should be carefully optimized. In addition, certain biological signals need to be internalized by cells in order to function effectively. In this case, our approach can be utilized to display specific binding ligands for proteins which will not require the modification of the proteins and will enable their internalization. Lastly, the number of possible on-off cycles enabled by our approach may differ for various cell types for several reasons. First, the dynamic switching is driven by toehold-mediated strand displacement, leading to dissociation of a double-stranded ‘waste’ product. The accumulation of ‘waste’ after several cycles may lead to a progressive loss in the switching efficiency. Finally, after extended time in culture, ECM matrix proteins secreted by the cells may limit the effect of the synthetic signals.

In conclusion, DNA conjugated to peptides and proteins has great potential in the control of interactions between cells and their matrix environment. Our platform addresses the challenge of controlling the dynamics of bioactivity on synthetic scaffolds, an essential feature of extracellular matrix biology. We expect this approach will uncover mechanistic information for crafting specialized environments that guide processes such as stem cell proliferation and differentiation. Use of the P-DNA conjugates can be extended from surfaces to bulk materials of any chemistry, thus rendering them dynamic systems for cell signalling. Furthermore, the building blocks of P-DNA degrade into naturally occurring structural units and therefore will easily clear after a finite period of time. The programmability of DNA will also allow probing the effect of many different signals independently, as well as their synergies. At the same time, coupling this capability to the mature field of DNA micropatterning affords additional opportunities, such as single-cell resolution and high-throughput screening to discover the behaviour of cells in their niche. Lastly, this platform has the potential to control dynamically the presentation of biological signals *in vivo*. By modifying an implanted soft biomaterial with DNA handles, signals could be added or removed by injecting small molecules and allowing them to interact with the biomaterials. Thus, it should be possible to tune the *in vivo* microenvironment dynamically.

## Methods

### Peptide synthesis and purification

The F^az^GGRGDS and F^az^GGRGES peptides (Supplementary Fig. 1a; F^az^ denotes the unnatural amino acid 4-azidophenylalanine), K^az^GGPHSRN peptide (Supplementary Fig. 9b; K^az^ denotes the unnatural amino acid azidolysine), K^az^GGIKVAV peptide (Supplementary Fig. 12a), and FGF mimetic peptide K^az^-GGYRSRKYSSWYVALKR (Supplementary Fig. 12a) were synthesized using standard fluoren-9-ylmethoxycarbonyl (Fmoc) solid-phase peptide synthesis on rink amide MBHA resin (100–200 mesh, 0.55 mmol g^−1^). Following synthesis, each peptide was cleaved from the resin in a 95:2.5:2.5 mixture of trifluoroacetic acid:triisopropyl silane:water, and precipitated using cold diethyl ether. The peptides were purified using reverse phase HPLC (Varian Prostar 363, Jupiter 10u Proteo 90A column) using a water/acetonitrile gradient (2–50% acetonitrile over 30 min) with 0.1% TFA. Purified peptides were lyophilized and stored at −20 °C. The purity of the peptides was confirmed by electrospray ionization mass spectrometry in positive mode (ESI-MS, Agilent 6510 Q-TOF).

### Synthesis purification and characterization of P-DNA conjugates

All P-DNA conjugates were synthesized according to the procedure shown in Supplementary Fig. 1b. Oligonucleotides modified with a terminal amine moiety were purchased from Integrated DNA Technologies and dissolved to 1 mM in 100 mM phosphate buffer, pH 8.5. To this solution was added dibenzocyclooctyne-sulfo-*N-*hydroxysuccinimidyl ester (DIBAC-sulfo-NHS, Sigma Aldrich) as a 100 mM solution in DMSO to a final concentration of 5 mM (5 eq., 5% total DMSO). The mixture was incubated for 2 h at room temperature, with vigorous shaking, after which an equivalent volume of 100 mM phosphate buffer, pH 9.5 was added. The mixture was exposed to a second aliquot of DIBAC-sulfo-NHS (5 eq.) and incubated for an additional 2 h at RT with vigorous shaking. Excess DIBAC was removed using a size exclusion column (Illustra NAP-5, GE Healthcare) pre-equilibrated with 20 mM phosphate buffer, pH 7.5. To this solution was added two volumes of the desired azide-containing peptide as a 1 mM solution in water to achieve a 2:1 molar ratio of peptide:DNA. Sodium chloride was added to a final concentration of 100 mM and the solutions were gently agitated overnight at RT (and 48 h for the FGF-2 peptide). Following reaction, samples were desalted using a NAP-5 column pre-equilibrated with 50 mM triethylammonium acetate (TEAA) buffer, pH 7. For the FGF-2 peptide, DMSO was added (20% total DMSO) to dissolve precipitates that formed during the conjugation reaction.

The P-DNA solutions were concentrated to ∼1 mM DNA using an Amicon Ultra-0.5 centrifugal filter unit, 3 kDa (Millipore) and purified using reverse phase HPLC (Agilent 1260 Infinity, DIKMA Inspire C18 column (5 um, 250 × 4.6 mm)) with a gradient of organic buffer B (90% acetonitrile in water, + 50 mM TEAA, pH 7) in water + 50 mM TEAA, pH 7 (buffer A). The gradient used was 5–50% B over 30 min. Absorbance at 260 nm was monitored, with unmodified DNA eluting at ∼12 min, and P-DNA conjugates approximately 17–20 min (Supplementary Fig. 1c). The P-DNA peak was collected, concentrated using an Amicon Ultra-0.5 centrifugal filter unit, 3 kDa, and lyophilized. Immediately before use, P-DNA aliquots were dissolved to the required concentration in 100 mM HEPES buffer, pH 7.5, and quantified by UV–Vis absorbance at 260 nm. The purity of the P-DNA conjugates was confirmed by electrospray ionization mass spectrometry in negative mode (ESI-MS, Agilent 6510 Q-TOF) and polyacrylamide gel electrophoresis using a 6% PAGE-gel (running time 60 min at 100 V).

### Preparation and characterization of P-DNA modified alginate surfaces

Alginate-coated glass surfaces were prepared as outlined in Supplementary Fig. 2a. Round coverslips (12 mm diameter, VWR Micro Cover Glasses) were cleaned by submerging them in 2% micro-90 solution (Sigma-Aldrich) in water at 60 °C for 30 min. After rinsing 6 times with water and 2 times with ethanol, the coverslips were dried and marked on one side to keep track of which side will be plasma etched. The coverslips were plasma etched for 10 min before dispersing them in 2% APTES solution in ethanol for 3 h. After the APTES coating, the coverslips were rinsed twice with ethanol and twice with water. To cure the APTES layer, the coverslips were loaded in an oven for 60 min at 120 °C. The APTES coated coverslips were stored at 4 °C for a maximum of 1 week.

A solution of alginate in ddH_2_O (0.5 wt%) was activated by dissolving 5 mg of 1-ethyl-3-(3-dimethylaminopropyl)carbodiimide (EDC) and 10 mg of *N*-hydroxysuccinimide (NHS) per ml of solution. The APTES-treated coverslips were soaked in the activated alginate for 1 h at RT. Unconjugated polymer was removed and the coverslips exposed to a second aliquot of activated alginate for 1 h at RT, followed by rinsing three times with double-deionized water (ddH_2_O). To couple the DNA to the alginate surface, an amine-modified surface strand (10 μM in 100 mM HEPES buffer, pH 7.5) was used to dissolve EDC and NHS to concentrations of 5 and 10 mg ml^−1^, respectively. 100 μl of this solution was dropped onto the APTES-treated coverslips and allowed to react for 1 h at RT, followed by rinsing three times with ddH_2_O.

The DNA modification of the alginate was confirmed by incubating the substrates with a fluorescein-labelled complementary strand, resulting in surface fluorescence (Supplementary Fig. 2b). Engineering a toehold into this fluorescent strand allowed its displacement, resulting in loss of fluorescence. The DNA-modified surfaces were incubated with the desired P-DNA conjugate (in 100 mM HEPES buffer, pH 7.5) for 1 h at RT, followed by washing three times with PBS. To determine the surface density of the bound signal, we used different concentrations of biotinylated complementary DNA to mimic the bioactive strand. The biotinylated surfaces were exposed to streptavidin-horseradish peroxidase (HRP) conjugate for 10 min at RT, then washed three times with PBS. The enzyme was assayed using 2,2′-azino-bis(3-ethylbenzothiazoline-6-sulphonic acid) (ABTS) and hydrogen peroxide monitoring the color evolution at 414 nm (Supplementary Fig. 2c), and the amount of HRP bound was derived by comparing to a standard curve of free enzyme. The relationship between applied concentration of the complementary strand and the resulting density of bioactive strands on the surface is shown in Supplementary Fig. 2d.

### Mechanical properties of the alginate layer

Rheological experiments were performed using Anton Paar MCR 302 rheometer with a 25 mM diameter 2° angle cone-plate oscillating geometry. Prior to testing, 100 μl of the alginate solution in ddH_2_O (1 wt%) was placed on the stage while 100 μl of gelling solution was placed on the upper fixture. Gelling solution contained 150 mM NaCl and either 0, 2, 4, 8, or 16 mM CaCl_2_. The fixture was lowered to the stage, diluting the alginate concentration to 0.5 wt%, the same concentration used to make the surface coatings. All experiments were performed at 37 °C. Prior to testing, the gap was sealed with mineral oil to prevent evaporation. Measurement began with a 15-minute equilibration period at 0.1% oscillatory strain and 10 s^−1^ oscillatory frequency. Next, a frequency sweep was performed (100 to 1 s^−1^) with the strain held at 0.1%. Finally, a strain sweep was performed from 0.1% to 100% strain at a constant 10 s^−1^ frequency. Three tests were performed for each CaCl_2_ concentration.

### Fibroblast adhesion assays

NIH-3T3 mouse embryonic fibroblasts (ATCC CRL-1658) were used as received without further authentication nor detection for mycoplasma contamination. Cells were cultured and passaged every 3 days in DMEM with high glucose supplemented with 10% fetal bovine serum (FBS) and 1% penicillin/streptomycin (P/S). For adhesion experiments 7,500 cells were plated on coverslips placed in 24-well plates in 0.5 ml of media and left to adhere for the desired time at 37 °C and 5% CO_2_. Following incubation, the cells were fixed with 4% paraformaldehyde (PFA) in PBS for 15 min at RT, then washed three times with PBS and incubated for 5 min with 0.4% Triton-X. The cells were then washed three times with PBS and stained with DAPI to visualize the nuclei and AlexaFluor-488 conjugated phalloidin to stain the actin filaments.

The coverslips were imaged using a TissueGnostics cell imaging and analysis system mounted on an upright microscope (TissueFaxs). Confocal micrographs of fluorescently stained samples were obtained using an inverted confocal laser scanning microscope (Zeiss LSM 510 META) or Nikon AR1 (see Imaging and cell analysis below). For the scanning electron micrograph images, cells were fixed with 2.5% glutaraldehyde in PBS (containing 1 mM CaCl_2_) for 1 h at room temperature. Fixed samples were dehydrated by exposure a graded series of water-ethanol mixtures. Once in 100% ethanol, samples were dried at the critical point of CO_2_ using a critical point dryer (Tousimis Samdri-795) to preserve structural details. Dried samples were then coated with 14 nm of osmium using an osmium plasma coater (Filgen, OPC-60A), and imaged using a Hitachi S-4800 Field Emission Scanning Electron Microscope working at an accelerating voltage of 5 kV.

### Cell viability

For cell viability experiments, media was exchanged with PBS containing 2 μM calcein-AM (Life Technologies) and 100 ng ml^−1^ propidium iodide (Sigma) for 20 min at 37 °C. The cells were then rinsed with PBS and imaged with an inverted epifluorescent microscope (Zeiss). Live and dead cells were counted using the Cell Counter plug-in for ImageJ.

### Antibody blocking experiments

Fibroblast cells cultures were treated with 10 μg ml^−1^ of α5, β1, β3 integrins, or combinations of α5/β 1, αv/β3 blocking antibodies and were analysed after 24 h. The antibodies used are: β1 integrin (Millipore AB1952); β3 integrin (abcam ab119992); α5 (abcam ab72663); α5 β3 (abcam ab78289).

### Cell adhesion dynamics and synergy experiments

For the RGDS adhesion experiments in Figs 1 and 2, alginate surfaces were modified with DNAsurf1 (surface strand, see Supplementary Table 1 for all DNA sequences), and then treated with 10 μM of bioactive strands 3’RGDS-DNA1 (in 100 mM HEPES, pH 7.5) for 1 h to allow hybridization. Unbound DNA was removed by rinsing three times with PBS. For the toehold and ExoIII systems, the DNA-RGDS bioactive strand was added as described above to achieve the ON state. To switch to the OFF state, the trigger (ssDNA at 10 μM for the toehold and hairpin, and 20 units of ExoIII (New England Biolabs) was added to the media and incubated with the cells for 3 h at 37 °C. The substrates and cells were then rinsed three times with media to remove any free DNA, peptides, or enzyme. To restore the ON state, a fresh aliquot of DNA-RGDS was added to the toehold and ExoIII systems, and a displacement strand to remove the opening strand for the hairpin system. For each data point shown in Fig. 2, an independent set of coverslips was fixed at the indicated time point, and cell area was quantified as described above.

To verify that all three dynamic systems are responding to their respective stimuli in the expected manner, the corresponding strands for each in solution were modified with a donor-quencher pair (fluorescein-BHQ) as shown in Supplementary Fig. 6d–f. For the toehold and ExoIII systems, the proximity of the donor and quencher in the ON state resulted in almost complete quenching of donor fluorescence (Supplementary Fig. 6g,i). For the hairpin system, the quenched state corresponds to the initial OFF state (Supplementary Fig. 6h). Adding the appropriate trigger to switch to the OFF state for the toehold and ExoIII systems, or the ON state for the hairpin system, separates the donor and quencher, resulting in robust donor fluorescence and confirming the dynamic switch of the system (Supplementary Fig. 6g–i). For each system, the component strands were mixed (10 μM each) and annealed from 80 °C to RT over 30 min. The donor fluorescence emission spectrum both before and 20 min after addition of the corresponding trigger (10 μM, toehold and hairpin; 20 units, ExoIII) was obtained on a Cytation 3 microplate reader (BioTek).

For the synergy experiments, separate substrates were modified with a surface strand bearing a different number *N* of nucleotides between two constant regions complementary to RGDS P-DNA and PHSRN P-DNA strands (Supplementary Fig. 9). The RGDS and PHSRN bioactive strands were added to a final concentration of 5 μM each in 100 mM HEPES buffer at pH 7.5, with 100 mM NaCl, and incubated for 1 h at RT to allow for hybridization to the surface strand. The surfaces were rinsed three times with PBS, followed by fibroblast adhesion and area quantification as described above. To determine the relationship between *N* and *d*, the RGDS and PHSRN peptides were replaced with fluorescein and TAMRA, respectively, as shown in Fig. 3b. The corresponding strands were mixed in solution (1 μM each), annealed from 80 °C to RT over 30 min, and the fluorescence emission spectrum (510–700 nm) upon donor excitation (at 495 nm) was obtained on a Cytation 3 microplate reader. The FRET efficiency of the two dyes in the different hybridized states was determined using equation (1). The quantum yields of FAM- and TAMRA-labelled DNAs were determined using monomeric FAM in aqueous solution as reference (QY=0.95) at an excitation wavelength of 495 nm. The overlap integral between the FAM emission spectrum and TAMRA absorption spectrum was calculated to be 3.2e^15^ M^−1^ cm^−1^ nm^4^, yielding a Förster distance of *R*_0_=57 Å. The distances were calculated from the measured FRET efficiencies by using equation 2.


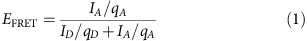



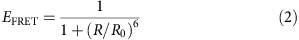


### BMP-2 modification and characterization

Recombinant human BMP-2 (rhBMP2), obtained from Medtronic Sofamor Danek, was modified with DNA in the two-step procedure shown in Supplementary Fig. 10a. To a solution of rhBMP2 dimer (50 μM in 10 mM phosphate buffer, pH 7.5) was added NHS-(PEG)_4_-azide (Thermo Scientific) in DMSO to a final concentration of 200 μM (2 eq.; <1% total DMSO) and vortexed briefly. The solution was incubated at RT for 2 h, and unreacted small molecule was removed by washing the solution three times with 10 mM citrate buffer (pH 3) using an Amicon Ultra-0.5 centrifugal filter unit, 3 kDa. To the retentate was added Y*_DNA_-DIBAC (2 eq. in water) and sodium chloride to 100 mM and the solution was incubated at RT overnight. Unreacted DNA was removed by washing the solution three times with 10 mM citrate (pH ∼3) using an Amicon Ultra-0.5 centrifugal filter unit, 10 kDa. The ratio of DNA versus protein was calculated as 0.9:1 using UV–Vis (Supplementary Fig. 10b), subtracting the spectrum of unmodified rhBMP2, and using the extinction coefficient of Y*_DNA_ (*ε*_260 nm_=262,100 M^−1^ cm^−1^) relative to that of rhBMP2 (*ε*_280 nm_=18,421 M^−1^ cm^−1^).

### BMP-2 activity assays with C2C12 cells

In order to tether both RGDS and rhBMP2 on the substrate, alginate was modified with two different surface strands, X_DNA_ and Y_DNA_ (5 μM of each in the coupling step), followed by hybridization of RGDS-X*_DNA_ (2 μM), BMP2-Y*_DNA_ (4 μM) or both, depending on the system. C2C12 mouse pre-myoblasts cells (ATCC CRL-1772) were used as received without further authentication nor detection for mycoplasma contamination. Cells were cultured and passaged at 37 °C, 5% CO_2_ every three days in DMEM containing 4.5 g l^−1^ glucose and 4 mM L-glutamine (ATCC), supplemented with 10% heat-inactivated FBS and 1% P/S. For differentiation experiments, cells from passage three to seven were used, and resuspended in low-serum media (DMEM, 0.5% FBS) prior to seeding on DNA surfaces. Cells were cultured for 4 days prior to either staining or quantitative assaying of alkaline phosphatase (ALP) activity. On day 4, ALP expression was determined as previously described (Lee, S. S. *et al*. *Adv Healthc Mater*. 4, 2015). The absorbance slope values from the ALP assay were normalized by the amount of dsDNA in each sample for the data shown in Fig. 4c.

### Animals

All animal housing and procedures were performed in accordance with the Public Health Service Policy on Humane Care and Use of Laboratory Animals and all procedures were approved by the Northwestern University Institutional Animal Care and Use Committee. Timed pregnant CD1 mice, were used for primary neural stem cells (NSC) cultures and were supplied by Charles River Laboratories (Wilmington, MA).

### NSC culture and assay

NSCs were derived from the spinal cord of newborn mice (P0), as described elsewhere^40^,^44^. Briefly, NSCs from spinal cord were isolated and mechanically dissociated using razor blades along with pipetting and grown in serum-free neurosphere growth medium (DMEM/F12 (Gibco/Invitrogen)) with N2 Supplement (Gibco/Invitrogen) and B27 Supplement (Gibco/Invitrogen), along with Pen/Strap/Glut 100 × Mix (Gibco/Invitrogen)) with EGF (20 ng ml^−1^, human recombinant, BD) plus bFGF (20 ng ml^−1^, human recombinant, Millipore) for 3–4 days, to generate neurospheres. Primary neurospheres were then passaged by dissociating with 0.05% trypsin (Invitrogen) for 2 min followed by incubation with a soybean trypsin inhibitor (Sigma), a 5-minute spin, and repeated trituration. Secondary and third passages were grown as described previously^44^ and used for subsequent studies.

### Neural stem cells adhesion and dynamic assays on P-DNA surfaces

Neurospheres were dissociated and plated at a density of 1 × 10^4^ cells per cm^2^ on surfaces coated with laminin (Roche); alginate; IKVAV, FGF-2 peptide or both peptides within 24-well culture plates, and then grown for 24, 48 and 72 h in neurosphere growth medium. To assess the influence of the different P-DNA platforms, cells were cultured without any growth factors. For the dynamic experiments with two signals, the substrates were modified with the DNA strands shown in Supplementary Fig. 6j. To switch to the OFF state, the trigger (ssDNA at 10 μM) was added to the media and incubated with the cells at 37 °C. The substrates and cells were then rinsed with media to remove any free DNA and peptides. To restore the ON state, a fresh aliquot of DNA-IKVAV or DNA-FGF-2 was added.

### Western blot/immunocytochemistry

For western blot analysis, protein extracts were obtained from the NIH-3T3 mouse embryonic fibroblasts and NSCs derived from murine spinal cord at different time points and total protein extracts were separated by SDS-polyacrylamide gel and electrotransferred to a nitrocellulose membrane (Bio-Rad) as described elsewhere (Alvarez *et al*. Biomaterials, 2014, 35,4769-81). Membranes were blocked with 5% bovine serum albumin (BSA, Sigma-Aldrich) and first incubated with primary antibodies overnight at 4 °C followed by their corresponding secondary HRP-conjugated antibodies (1:3,000; Thermo Fisher). Protein signal was detected using the ECL chemiluminescent system (Bio-rad). Densitometry analysis, standardized to Actin or GADPH as a control for protein loading, was performed using ImageJ software. For quantification, triplicate samples were analysed.

For immunofluorescence we followed the protocol described previously (Alvarez *et al*. Cereb. Cortex, 2016, 26, 1046–1058). Briefly, fixed samples (4% PFA for 15 min at RT) were incubated with primary antibodies and appropriate Alexa 488, Alexa 555 and/or Alexa 647 secondary antibodies (1:500, Molecular Probes). Phalloidin was used to stain F-actin (1:2,000, Sigma-Aldrich,) mouse anti-Vinculin (1:1,000, Sigma Aldrich) was used to stain focal adhesions and DAPI (1:500, Molecular Probes) to stain nuclei. Finally, the preparations were coverslipped with Mowiol (Calbiochem) for imaging.

The following primary antibodies were used for western blot and/or Immunocytochemistry; rabbit anti-GFAP (NSC, mature/reactive glial marker, 1:500–1:8,000, Dako Z0334), mouse anti-Nestin (NSC and radial glial marker, 1:250, Abnova Corporation MAB353), mouse anti-Tubulin III (Tuj-1, neuronal marker 1:10,000, Covance 802001), mouse anti-β1 (integrin marker, 1:250, millipore AB1952), mouse anti-β3 (integrin marker, 1:250, abcam ab119992), mouse anti-phospho-FGFR1 (FGF-2 receptor 1:10,000, abcam ab111124), mouse anti-Vinculin (Sigma Aldrich V9131), rabbit anti-Laminin (ECM protein, 1:1,000, sigma Aldrich L9393), rabbit anti-phospho-ERK1/2 (Kinase, 1:2,000, Abcam 47339), Sox-2 (NSC marker, 1:500, abcam ab79351), rabbit anti-α-5/β3 (integrin marker, 1:250, abcam ab78289), rabbit anti-Fibronectin (ECM protein, 1:200, abcam ab2413), rabbit anti-Ki67 (proliferation marker, 1:500, abcam ab66155), rabbit anti-phospho-FAK (kinase marker, 1:1,000,Cell Signaling 3281P), rabbit anti-Actin (1:1,000, Sigma Aldrich A3853) and mouse anti-GADPH (1:1,000, Cell Signaling 97166).

### Imaging and cell analysis

For video time-lapse analysis, cells were placed in a Nikon BioStation IM-Q time-lapse imaging system with a 1.3-megapixel cooled monochrome camera at 37 °C with 5% CO_2_. Cells were imaged in phase contrast. Pictures were taken every 1–4 min for 3 h for fibroblasts and every 1–6 min for 24 h to 6 days *in vitro* for NSCs. Cell displacement, speed and trajectory were calculated with the aid of the ‘Manual Tracking’ plug-in of the ImageJ software (National Institutes of Health, USA).

Bright field images were taken throughout experiments with a digital camera (Nikon). Fluorescent preparations were viewed and micrographs were captured with a Nikon A1R confocal laser-scanning microscope with GaAsP detectors. Images were assembled in Adobe Photoshop (v. 7.0), with adjustments for contrast, brightness and color balance to obtain optimum visual reproduction of data. Morphometric, quantitative, live-image analysis and migration assays were performed using ImageJ software (National Institutes of Health, USA).

Confocal images were reconstructed by the Imaris program (version 8.1, Bitplane scientific software) for 3D interactive data viewing with normal or shadow projections of cells screened under Nikon A1R confocal laser-scanning microscope with GaAsP detectors.

For quantification of fluorescently stained samples, a minimum of 190 randomly selected cells from a minimum of three independent batches of culture were included for each condition.

### Statistical analysis

Statistical analysis was performed using Graphpad Prism v.6 software. Analysis of variance (ANOVA) with a Bonferroni *post hoc* test was used for all multiple group experiments and equality of variances was confirmed by Levene’s Test.

*P* values<0.05 were deemed significant. Values in graphs are shown as mean±s.e.m.

### Data availability

All data generated or analysed during this study are included in this published article (and its Supplementary Information files) and/or are available from the corresponding author on reasonable request.

## Additional information

**How to cite this article:** Freeman, R. *et al*. Instructing cells with programmable peptide DNA hybrids. *Nat. Commun.* **8**, 15982 doi: 10.1038/ncomms15982 (2017).

**Publisher’s note:** Springer Nature remains neutral with regard to jurisdictional claims in published maps and institutional affiliations.

## Supplementary Material

Supplementary Information Supplementary Figures and Supplementary Tables.

Supplementary Movie 1 Fibroblasts grown on RGDS surface. Live imaging of 3T3 fibroblast cells on RGDSmodified surface. Images were captured once every 3 minutes using a Nikon BioStation IM-Q imaging system with a 1.3-megapixel cooled monochrome camera (40 × len) and processed using the Image J program. Total time = 05h 57min. Scale bar: 10μm.

Supplementary Movie 2 Fibroblasts grown on alginate surface. Live imaging of 3T3 fibroblast cells on alginatecoated surface. Images were captured once every 4 minutes using a Nikon BioStation IM-Q imaging system with a 1.3-megapixel cooled monochrome camera (40 × len) and processed using the Image J program. Total time = 05h 50min. Scale bar: 10μm.

Supplementary Movie 3 Fibroblasts grown on RGDS surface. Live imaging of 3T3 fibroblast cells on RGDSmodified surface. Images were captured once every 2 minutes using a Nikon BioStation IM-Q imaging system with a 1.3-megapixel cooled monochrome camera (20 × len) and processed using the Image J program. Total time =11h 32min. Scale bar: 40μm.

Supplementary Movie 4 Fibroblasts grown on alginate surface. Live imaging of 3T3 fibroblast cells on alginatecoated surface. Images were captured once a minute using a Nikon BioStation IM-Q imaging system with a 1.3-megapixel cooled monochrome camera (20 × lens) and processed using the Image J program. Total time = 05h 51min. Scale bar: 40μm.

Supplementary Movie 5 Dynamic switching of RGDS. Live imaging of 3T3 fibroblast cells on RGDS P-DNA surface during an ON-OFF cycle. Images were captured once every 2 minutes using a Nikon BioStation IM-Q imaging system with a 1.3-megapixel cooled monochrome camera (20 × lens) and processed using the Image J program. Total time = 6h 43min. Scale bar: 10μm.

Supplementary Movie 6 Neural stem cells on IKVAV-modified surface after plating. Live imaging shows a neurosphere (NSC) on a P-DNA surface presenting IKVAV during 24h in vitro. Images were captured once every 6 minutes using a Nikon BioStation IM-Q imaging system with a 1.3-megapixel cooled monochrome camera (20 × lens) and processed using the Image J program. Total time = 23h 12min. Scale bar: 20μm.

Supplementary Movie 7 Neural stem cells on IKVAV-modified surface after 30h in culture. Live imaging shows a neurosphere on a P-DNA surface presenting IKVAV from 30 to 48h in vitro. Images were captured every 2 minutes using a Nikon BioStation IM-Q imaging system with a 1.3-megapixel cooled monochrome camera (20 × lens) and processed using the Image J program. Total time = 18h 32min. Scale bar: 20μm

Supplementary Movie 8 Switching OFF the IKVAV peptide signal (1). Live imaging of a neurosphere after removing (switch OFF) the IKVAV signal. Images were captured once every 4 minutes using a Nikon BioStation IM-Q imaging system with a 1.3-megapixel cooled monochrome camera (20 × lens) and processed using the Image J program. Total time = 34h. Scale bar: 20μm

Supplementary Movie 9 Switching OFF the IKVAV peptide signal (2). Live imaging of a neurosphere after removing (switch OFF) the IKVAV signal. Images were captured once every 4 minutes using a Nikon BioStation IM-Q imaging system with a 1.3-megapixel cooled monochrome camera (20 × lens) and processed using the Image J program. Total time = 34h. Scale bar: 20μm

Supplementary Movie 10 Dynamic switching of IKVAV over several cycles. Live imaging of a neurosphere upon switching the IKVAV signal ON and OFF repeatedly. Images were captured once every 6 minutes using a Nikon BioStation IM-Q imaging system with a 1.3-megapixel cooled monochrome camera (20 × lens) and processed using the Image J program. Total time = 107h 57min (4days 11h 57min). Scale bar: 30μm

Supplementary Movie 11 Dynamic switching of two peptide signals sequentially. Live imaging of a neurosphere grown on a FGF-2-modified surface (FGF ON) followed by the sequential addition and removal of the IKVAV signal (IKVAV ON and OFF). Images were captured once a minute using a Nikon BioStationIMq imaging system with a 1.3-megapixel cooled monochrome camera (40 × lens) and processed using the Image J program. Total time = 32h 14min. Scale bar: 10μm

Supplementary Movie 12 Dynamic switching of two peptide signals independently (1). Live imaging of a neurosphere on a surface presenting both FGF-2 and IKVAV peptide signals and dynamically switching each signal OFF and ON. Images were captured once every 6 minutes using a Nikon BioStationIMq imaging system with a 1.3-megapixel cooled monochrome camera (40 × lens) and processed using the Image J program. Total time = 107h 57min (4days 11h 57min). Scale bar: 30μm.

Supplementary Movie 13 Dynamic switching of two peptide signals independently (2). Live imaging of a neurosphere on a surface presenting both FGF-2 and IKVAV peptide signals and dynamically switching each signal OFF and ON. Images were captured once every 6 minutes using a Nikon BioStationIMq imaging system with a 1.3-megapixel cooled monochrome camera (40 × lens) and processed using the Image J program. Total time = 107h 57min (4days 11h 57min). Scale bar: 30μm.

Supplementary Movie 14 Neural stem cells on laminin-coated surface. Live imaging of a neurosphere on a laminin-coated surface. Images were captured once every 6 minutes using a Nikon BioStation IM-Q imaging system with a 1.3-megapixel cooled monochrome camera (20 × lens) and processed using the Image J program. Total time = 40h 12min. Scale bar: 20μm.
