# Supplementary material for: Instructing cells with programmable peptide DNA hybrids
Source: Nat Commun. 2017 Jul 10;8:15982. doi: 10.1038/ncomms15982 (PMC5508132; doi:10.1038/ncomms15982)
Supplement: Supplementary Information — Supplementary Figures and Supplementary Tables. [file ncomms15982-s1.pdf]

Supplementary Information

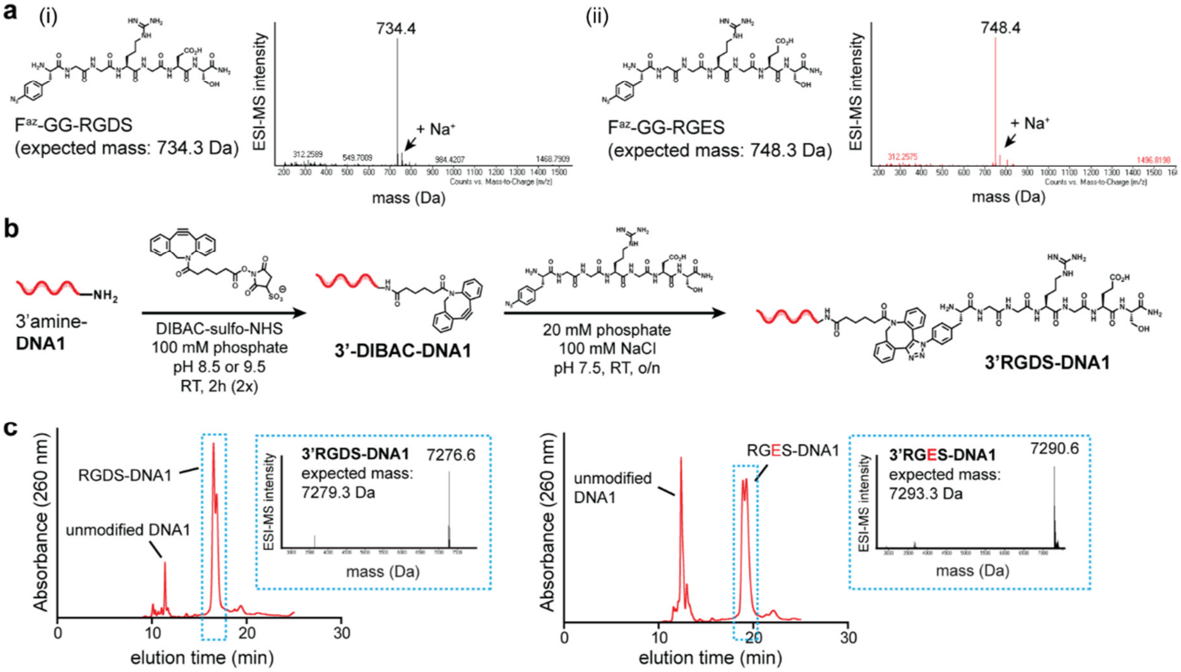

Supplementary Fig. 1: Synthesis and characterization of P-DNA conjugates. a) Chemical structures and mass spectra of the RGDS (a(i)) and RGES (a(ii)) peptides. Two glycine residues were introduced as a spacer, along with a 4-azidophenylalanine (F^{az}) residue for click coupling to DNA. **b)** Synthetic scheme for the P-DNA conjugate. Single-stranded DNA bearing an amine moiety was reacted with DIBAC-sulfo-NHS to install the cyclooctyne for subsequent copper-free click coupling to the azide-containing peptide (e.g. RGDS here). **c)** Reverse-phase HPLC traces of the RGDS-DNA and RGES-DNA conjugates, respectively. The P-DNA peaks can be separated from the unmodified DNA, and their purity verified by ESI-MS. No unmodified DNA (mass = 6228.2 Da) was observed in the purified peaks.

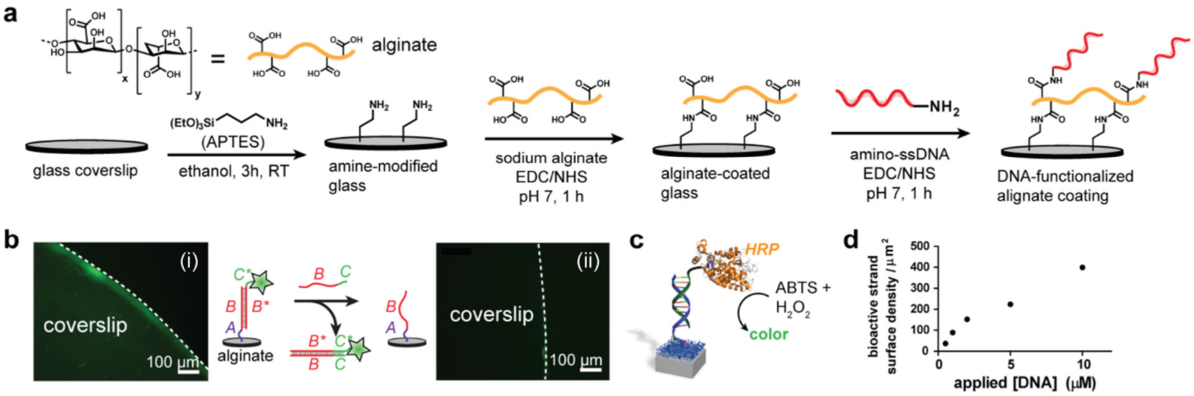

Supplementary Fig. 2: Surface modification and characterization. a) Synthetic protocol for coupling alginate to glass substrates. **b)** The modification of the alginate surface was probed using a fluorescent complement to the surface strand. Adding the complement resulted in a fluorescent surface (b(i)), and removing it via toehold-mediated strand displacement abolished any fluorescence (b(ii)). **c)** The amount of DNA on the surface was quantified by using an HRP-modified complementary strand, allowing colorimetric quantification of the number of bound DNA strands. **d)** Relationship between the applied concentration of complementary strand and the number of strands bound per square micrometer. This result demonstrate that our synthetic matrices are designed to display relatively dilute densities of epitopes that are spaced by several nanometers thus mimicking roughly signal density display in natural systems. For example, Fibronectin polymers in a natural matrix contribute one epitope per protein copy that measures several nanometers. Therefore, we do not expect great disparity in the signal densities between natural matrices and those of the synthetic matrices we are interested in for biological signaling.

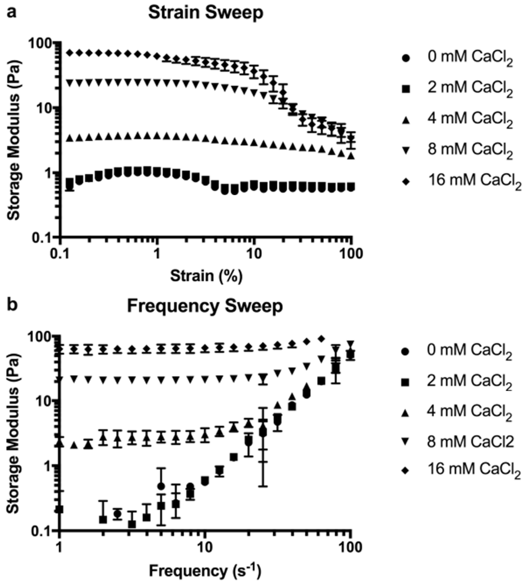

Supplementary Fig. 3: Mechanical properties of the alginate layer. a) Storage modulus of alginate gels in the presence of increased $CaCl_2$ concentration as a function of **a,** strain, **b)** frequency. Gels were formed by mixing 100 µL of 1 wt% alginate and 100 µL of gelling solution comprising 150 mM NaCl and the listed concentration of $CaCl_2$. Frequency sweeps were performed at 0.1 % strain and strain sweeps were performed at 10 s^{-1} frequency. These results demonstrate that the elastic modulus of the alginate layer is similar to that of soft tissues such as the brain (Science, 2009, 324, 1673-1677).

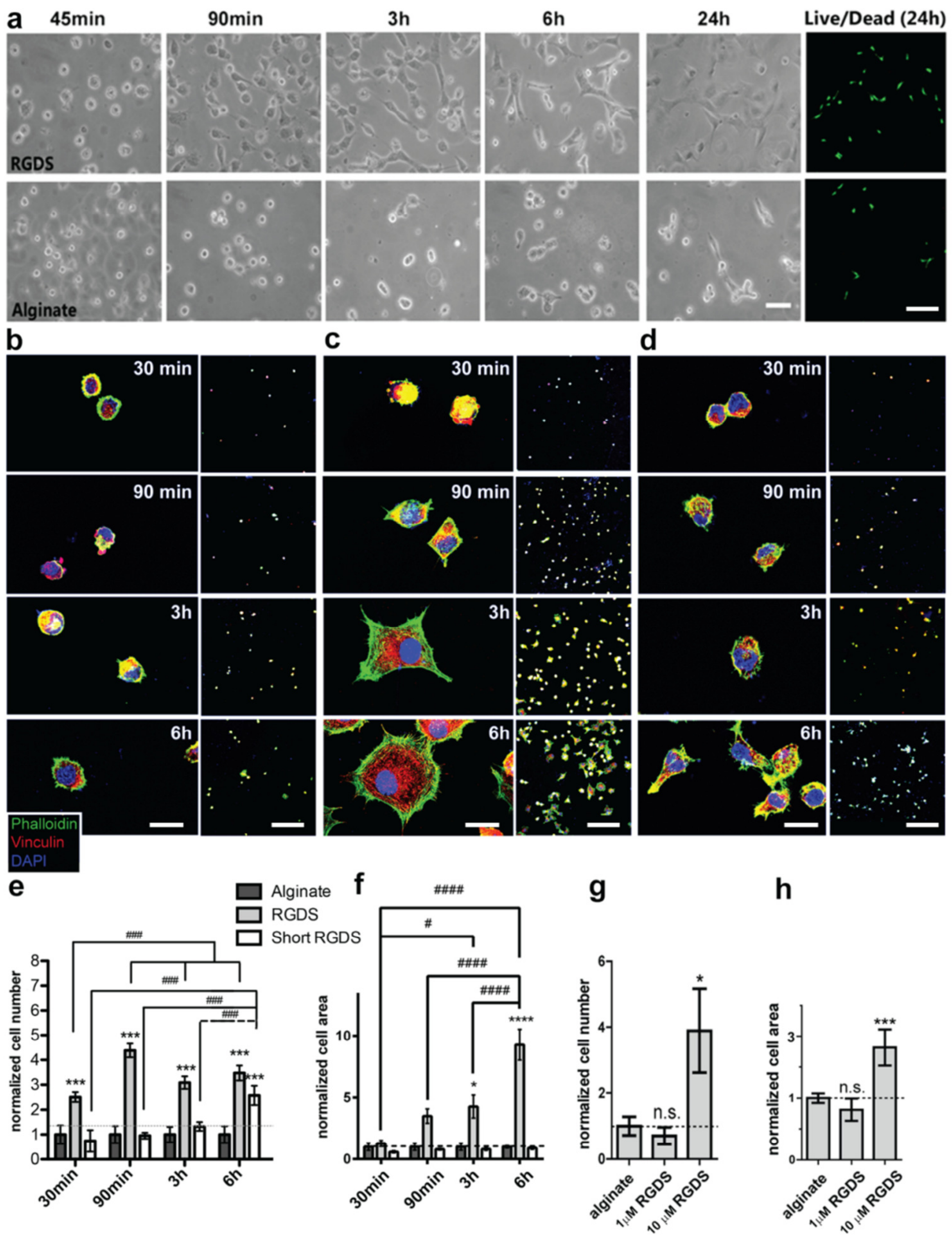

Supplementary Fig. 4: The effect of incubation time, ligand availability and RGDS concentration on the adhesion and spreading of fibroblast cells. a) Phase-contrast images of cells on indicated surfaces at different time points and calcein/PI staining of cells on RGDS and alginate surfaces at 24 hr. Scale bars: 10 µm for phase contrast, 100 µm for fluorescent images and relevant to all images respectively. **b-d)** Confocal images of cells at various time points on surfaces modified with alginate, RGDS with a longer DNA linker (DNAsurf1), and RGDS with a shorter DNA linker (GGCTGTCCACTGAG), respectively. Staining for actin (phalloidin, green), vinculin (red) and (DAPI, blue) reveal the cytoskeletal organization, focal adhesions and cell nuclei, respectively. Scale bars: 10 µm (zoom in images), 100 µm (wide field images). Scale bars relevant to all images in a column. **e,f)** Normalized cell number and area on indicated surfaces (values in each time point are relative to alginate. **g,h)** Normalized cell number and area on surfaces modified with varying concentrations of RGDS. (all values relative to alginate) (* $p < 0.05$, ** $p < 0.01$, *** $p < 0.001$, **** $p < 0.0001$).

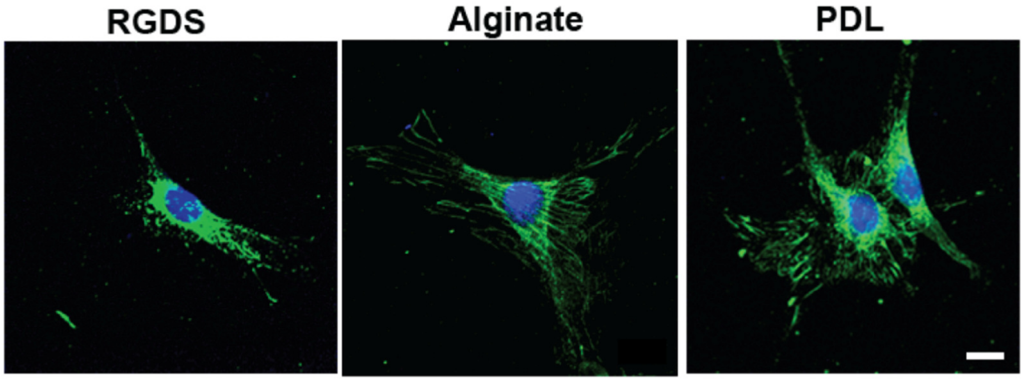

Supplementary Fig. 5: Fibronectin secretion by cells on various surfaces. Confocal images of fibroblasts cells after 24 hr on surfaces modified with a) RGDS b) Alginate c) Poly-D-Lysine. Staining for fibronectin (green) reveal the fibrillar matrix formation on the different substrates. Scale bar: 10 µm, relevant to all images.

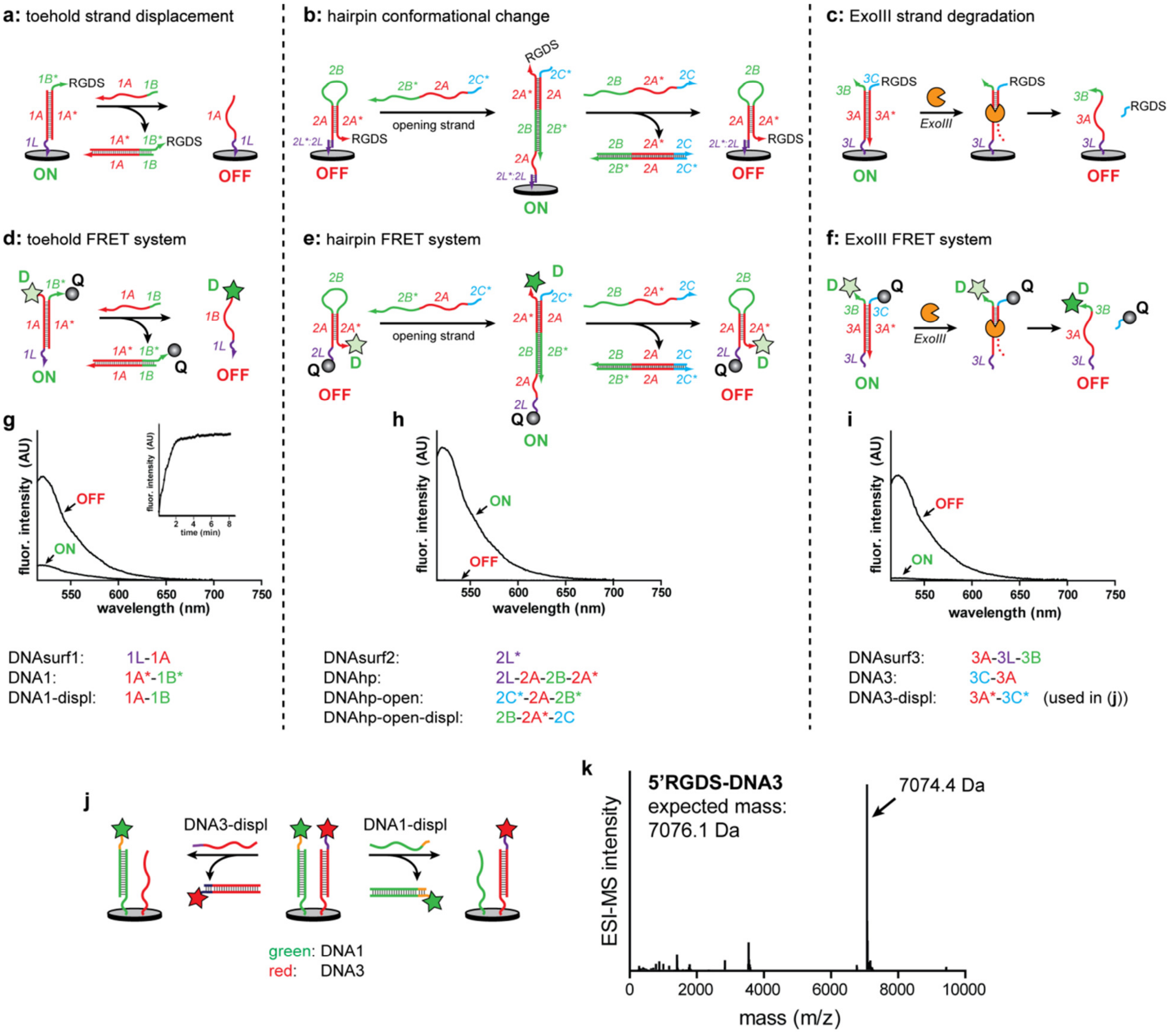

Supplementary Fig. 6: Design and characterization of dynamic RGDS systems. a-c) Strand design for switching of toehold-mediated strand displacement, hairpin conformational change, and ExoIII cleavage systems, respectively. Stars denote complementary sequences, and strand names and letters correspond to the sequences illustrated in Extended Data Table 1. **d-f)** Corresponding FRET systems for testing toehold-mediated strand displacement, hairpin conformational change, and ExoIII cleavage systems, respectively. In all cases, a donor dye (fluorescein) was attached to one strand, and a quencher (BHQ) to the other. Displacement, conformational change, or degradation of the constructs resulted in donor fluorescence, as verified by the fluorescence emission graphs. **g**-**i**) Emission spectra of the donor fluorophores in the corresponding states indicated in **d**-**f**. Note: in all cases, **OFF** and **ON** correspond to the relative accessibility of RGDS as described in Figure 2, not the emission of the dye. **g inset**, time-dependent fluorescence increase of the donor dye (at 520 nm) upon addition of the displacement strand. **j)** Schematic of the two-color system depicted in Figure 2j,k with two orthogonal dynamic triggers. **k)** ESI mass spectrum of the 5'RGDS-DNA3 conjugate used in the ExoIII system and the two-color experiment.

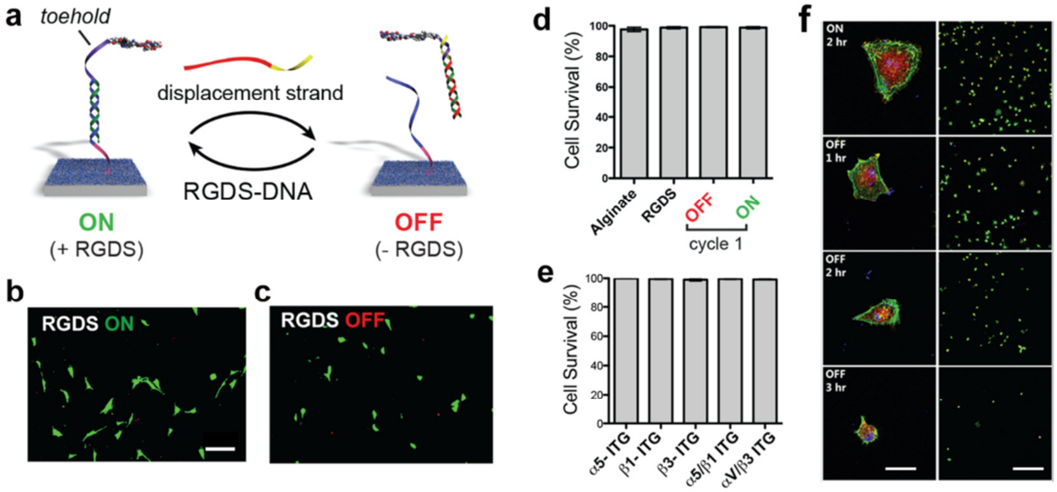

Supplementary Fig. 7: Cell survival assays on various surfaces and characterization of the 'switching off' step. a) Schematic of toehold mediated strand displacement system. Engineering a toehold into the bioactive strand allows its removal by a fully complementary displacement strand, thus switching bioactivity **OFF**. This process regenerates the surface strand, allowing for a second addition of the RGDS-DNA to turn bioactivity back **ON**. This process can be repeated for multiple cycles. **b,c)** Live/dead assay using calcein (green)/PI(red) staining of cells on surfaces with RGDS toggled between **ON** and **OFF**. Scale bar: 100 µm, relevant to both images. **d,e)** Percentage of cell survival on indicated surfaces or following treatment with integrin-blocking antibodies. **f)** Confocal images of fibroblasts cells at various time points after switching off the RGDS signal. Staining for actin (phalloidin, green), vinculin (red) and (dapi, blue) reveal the cytoskeletal organization, focal adhesions and cell nuclei, respectively. Scale bars: 10 µm (for zoom-in column) and 100 µm (for wide-field column).

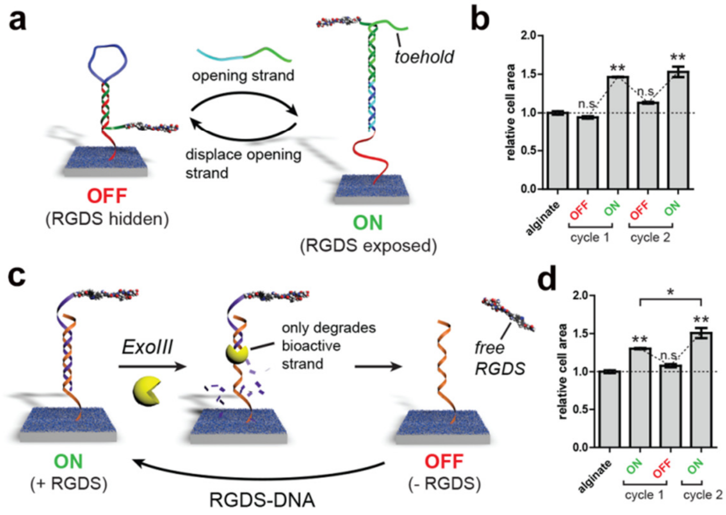

Supplementary Fig. 8: Additional mechanisms for dynamic, reversible switching of cell bioactivity. a) Schematic of reversible presentation of the RGDS signal by switching a DNA hairpin between the closed (**OFF**) and open (**ON**) states using opening and displacement strands. **b)** Cell area of fibroblasts plated on alginate surfaces, or on surfaces switched between two states shown in (**a**). **c)** Schematic of the ExoIII-mediated degradation of the bioactive strand, switching between the **ON** and **OFF** states using the enzyme. **d)** areas of fibroblasts plated on alginate surfaces, or on surfaces switched between two states shown in (**c**) For all samples, the time between successive **ON** and **OFF** state was 3 h. (* $p < 0.05$; ** $p < 0.01$).

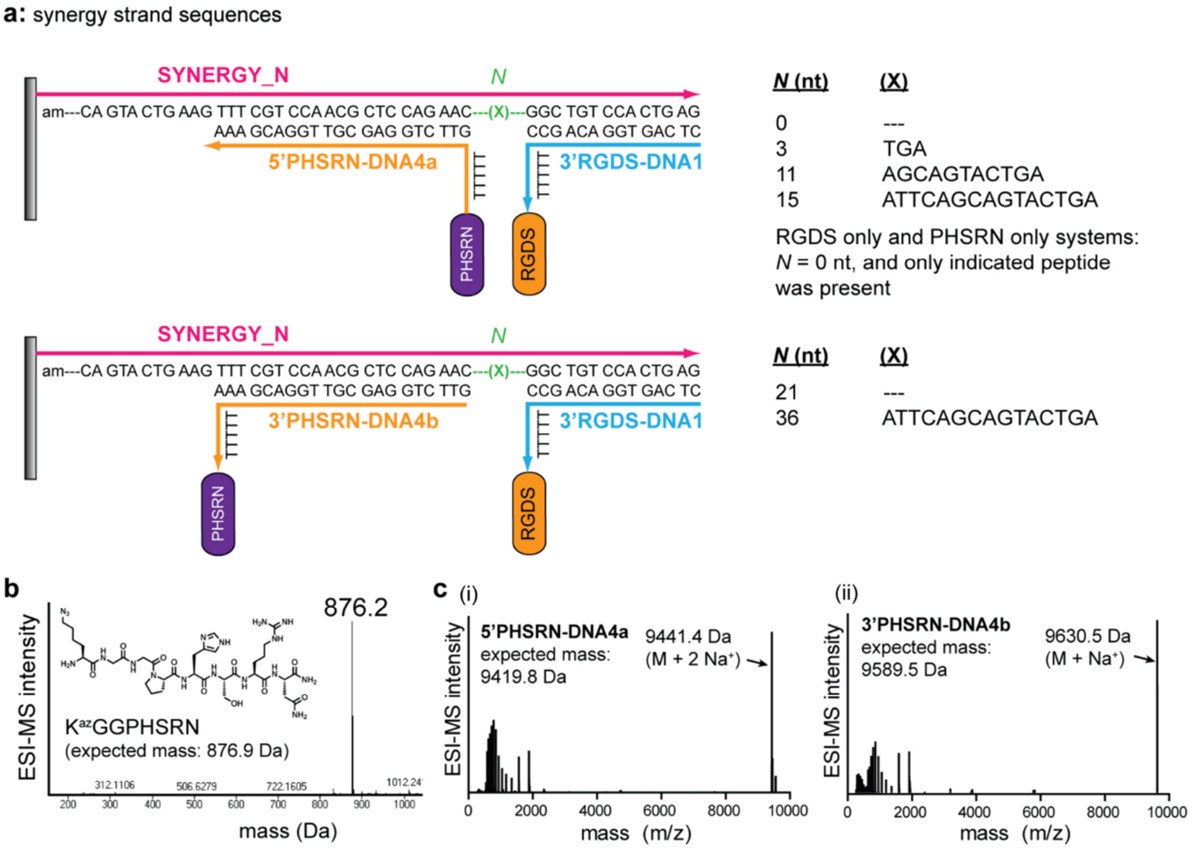

Supplementary Fig. 9: Design and characterization of the RGDS/PHSRN synergy system. a) Design and sequences of the strands used to controlling the spacing of the RGDS and PHSRN epitopes. For the RGDS and PHSRN only systems, *N* was zero nucleotides, and only the indicated peptide was present. **b)** Chemical structure and mass spectrum of the PHSRN peptide used. Two glycine residues were introduced as a spacer, along with an azidolysine (K^{az}) residue for click coupling to DNA. **c)** ESI-MS spectra of PHSRN-DNA4a (i) and PHSRN-DNA4b (ii) conjugates used for the synergy experiments.

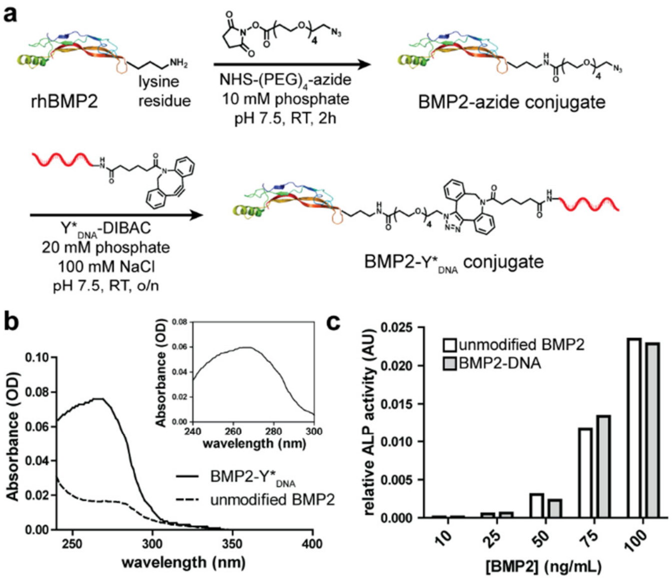

Supplementary Fig. 10: Synthesis and characterization of DNA-modified BMP-2. a) rhBMP-2 was conjugated to DNA by first modifying lysine residues with an NHS-(PEG)$_4$-azide linker, then exposing the protein to DNA-DIBAC for the copper-free click reaction. **b)** UV-Vis spectrum of the unmodified rhBMP-2 and the DNA-modified BMP-2. The increase in the absorbance at 260 nm due to the DNA is clearly visible. Inset: the difference between the modified and unmodified spectra, highlighting the DNA absorbance. **c)** ALP activity assay of the unmodified vs. DNA-modified BMP-2. The proteins are equally active across a range of concentrations, demonstrating that the conjugated DNA does not reduce the bioactivity.

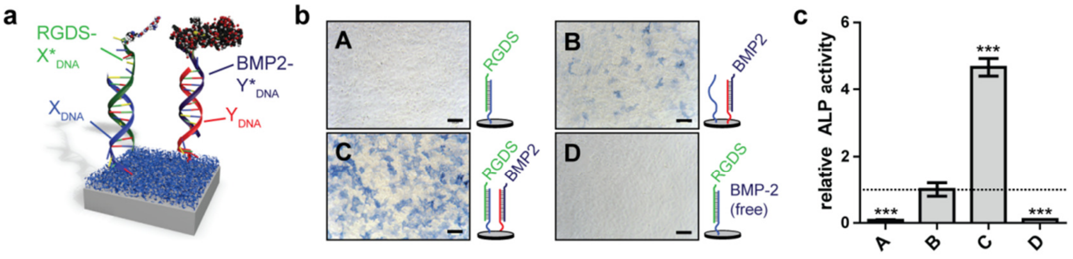

Supplementary Fig. 11: Synergy between RGDS and BMP-2 on DNA-modified surfaces. a) Schematic representation of surfaces modified with immobilized RGDS and BMP-2 using two different DNA strands. **b)** C2C12 cells were cultured on surfaces with bioactive strands presenting: RGDS only (**A**); BMP-2 only (**B**); RGDS and BMP-2 (**C**); RGDS and free BMP-2 in solution (**D**), and stained to visualize ALP expression. Scale bars for all images 100 µm. **c)** Quantitative ALP activity, relative to system **B** (values normalized by cell number). Systems **B**, **C**, and **D** all contain the same total amount of BMP-2. (*** $p < 0.001$).

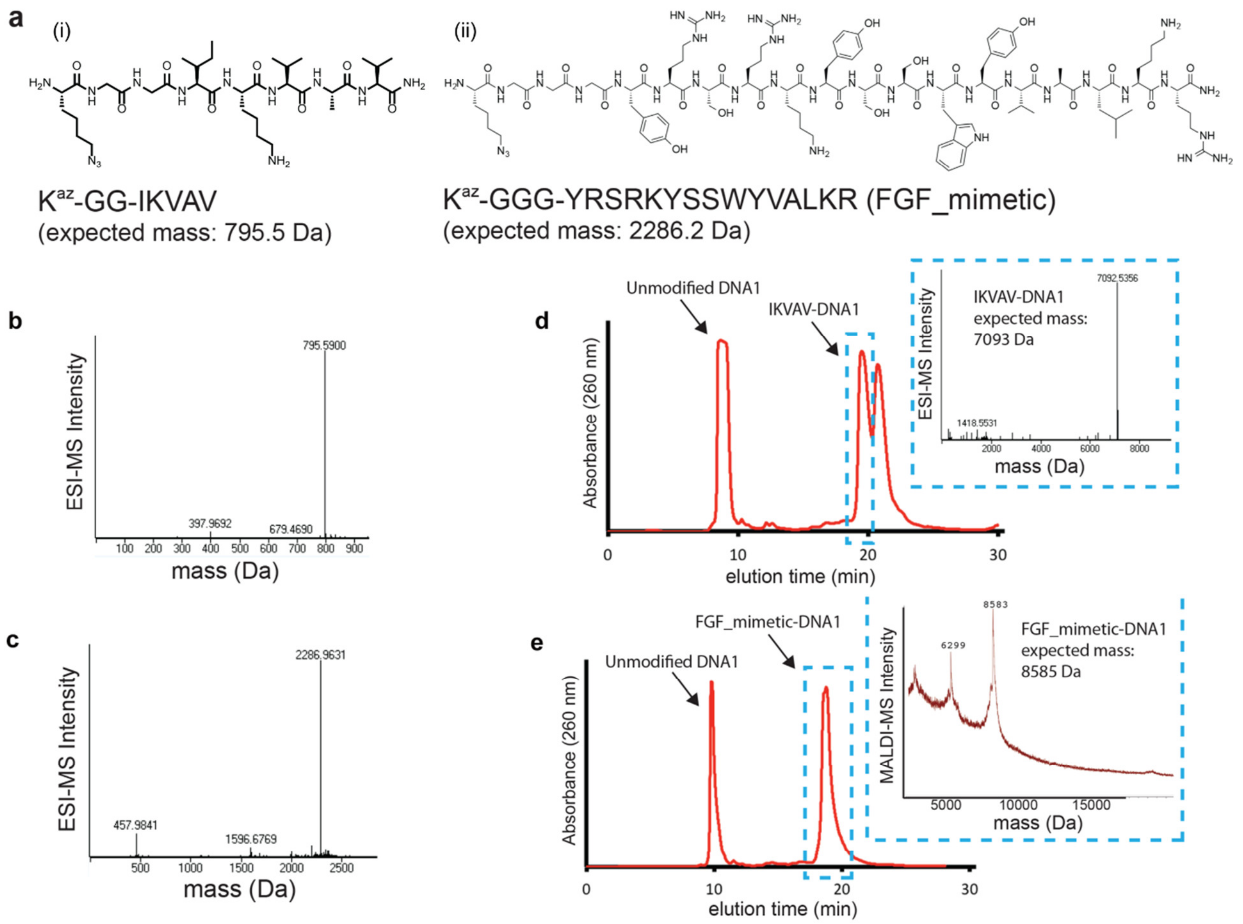

Supplementary Fig. 12: Synthesis and characterization of IKVAV and FGF-2 P-DNA conjugates. a) Chemical structures and mass spectra of the IKVAV (a(i)) and FGF-2 (a(ii)) peptides. Two glycine residues were introduced as a spacer, along with an azidolysine (K^{az}) residue for click coupling to DNA. **b,c)** ESI mass spectra for the IKVAV and FGF-2 peptides, respectively. **d,e)** Reverse-phase HPLC traces of the IKVAV-DNA and FGF2-DNA conjugates, respectively. The P-DNA peaks can be separated from the unmodified DNA, and their purity verified by ESI-MS (inset).

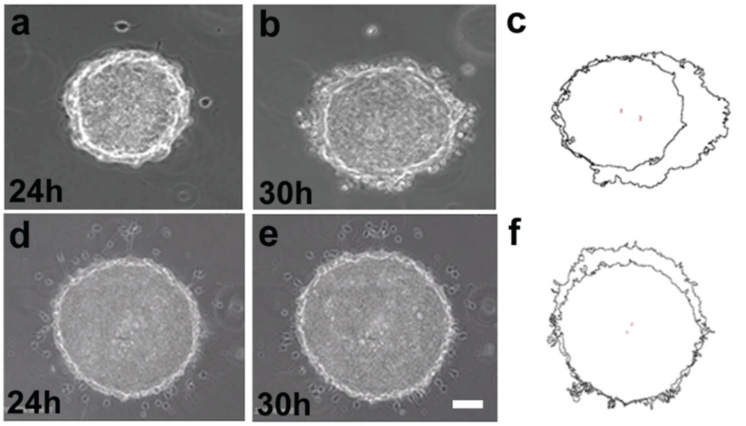

Supplementary Fig. 13: Neurospheres grown on surfaces modified with the FGF-2 peptide signal. a-b) Bright-field time-lapse images of a neurosphere grown on a surfaces modified with the FGF-2 mimetic peptide after 24 hours (a) and 30 hours (b). **c)** Outline of entire neurosphere at the different time-points. **d-e)** Bright-field time-lapse images of a neurosphere grown on a surfaces modified with the FGF-2 mimetic peptide after 24 hours (d) and 30 hours (e). **f)** Outline of entire neurosphere at the different time-points. Scale bar: 10 µm, relevant to all images.

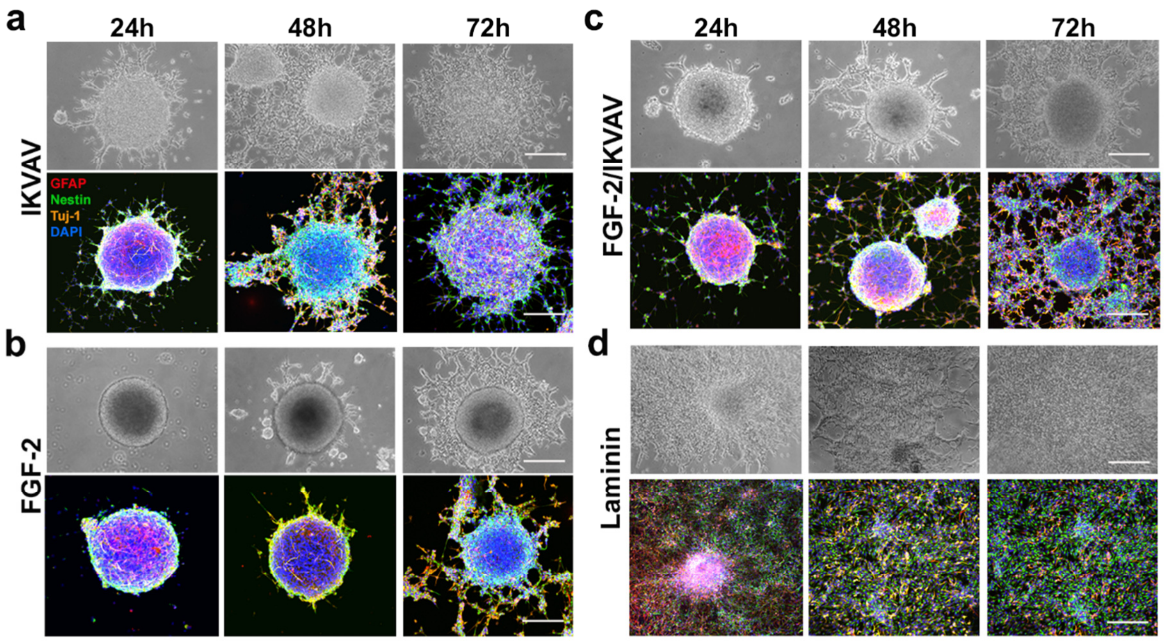

Supplementary Fig. 14: Neurospheres grown on surfaces modified with various peptide signals. Bright field and confocal images of neural stem cells taken at different time points on surfaces modified with **a)** IKVAV **b)** FGF-2 **c)** both FGF-2 and IKVAV **d)** Laminin control. All scale bars: 50 µm, relevant to all images in a row.

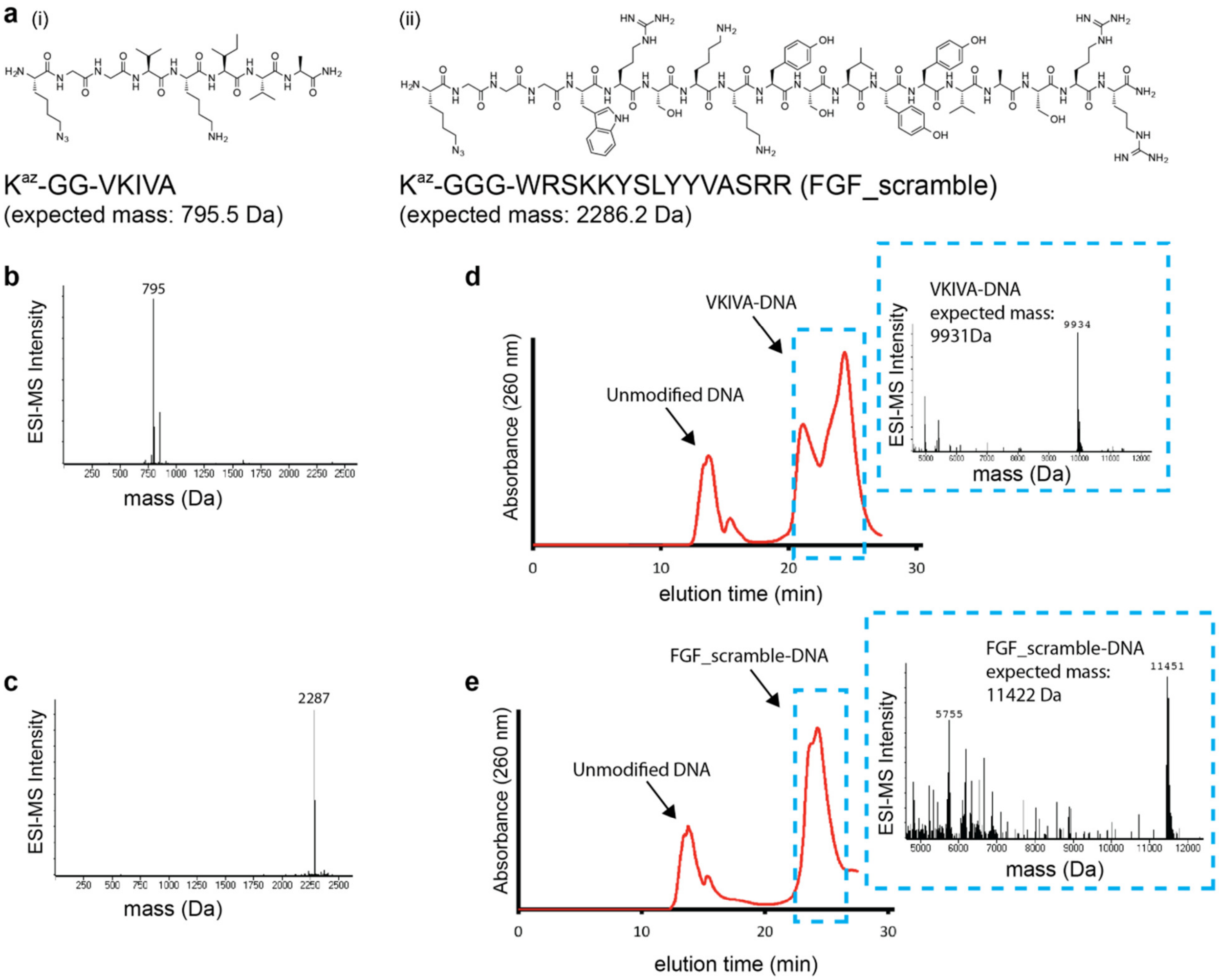

Supplementary Fig. 15: Synthesis and characterization of scrambled IKVAV and scrambled FGF-2 P-DNA conjugates. **a)** Chemical structures and mass spectra of the scrambled IKVAV (a(i)) and scrambled FGF-2 (a(ii)) peptides. Two/three glycine residues were introduced as a spacer, along with an azidolysine (K^{az}) residue for click coupling to DNA. **b,c)** ESI mass spectra for the scrambled IKVAV and scrambled FGF-2 peptides, respectively. **d,e)** Reverse-phase HPLC traces of the scrambled IKVAV-DNA and scrambled FGF2-DNA conjugates, respectively, and their purity verified by ESI-MS (inset).

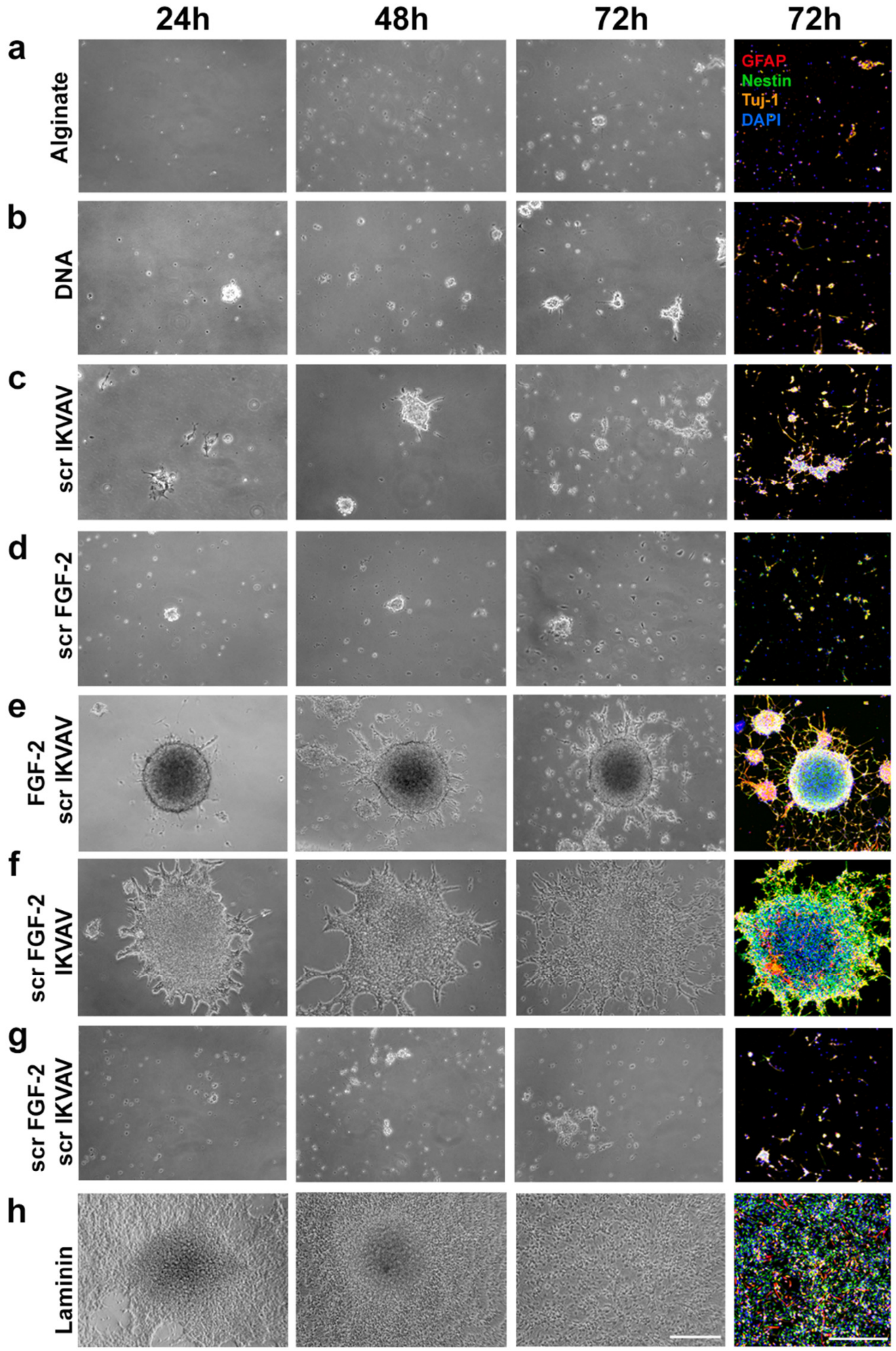

Supplementary Fig. 16: Monitoring the specificity of the bioactive peptide signals. Bright field and confocal images of neural stem cells taken at different time points on surfaces modified with **a)** alginate **b)** single stranded DNA **c)** scrambled IKVAV **d)** scrambled FGF-2 **e)** FGF-2 and scrambled IKVAV **f)** scrambled FGF-2 and IKVAV **g)** scrambled FGF-2 and scrambled IKVAV **h)** Laminin positive control. All scale bars: 50 µm, scale bars relevant for all grayscale images in a row.

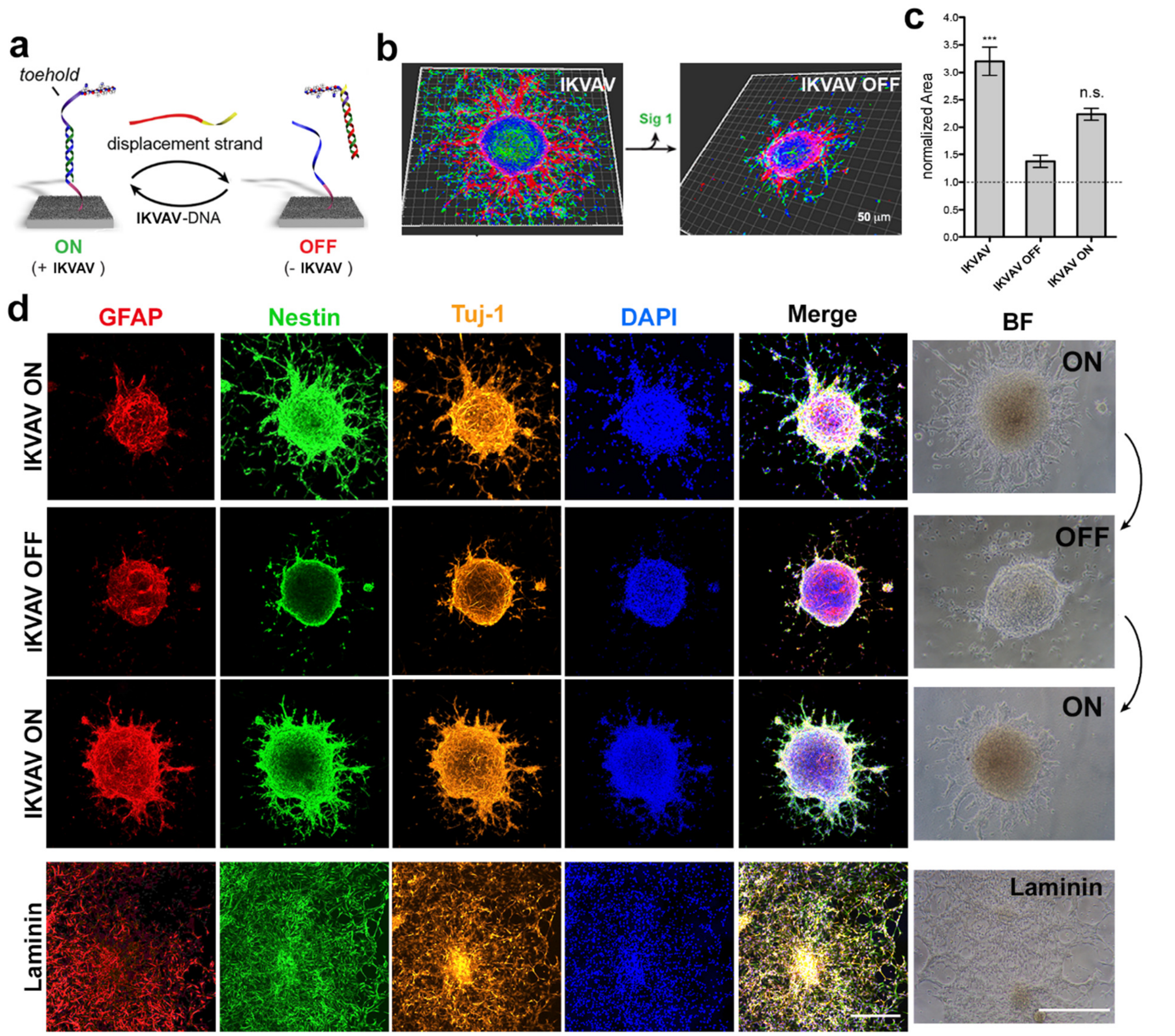

Supplementary Fig. 17: Effect of Dynamic and reversible presentation of IKVAV on NSC culture. a) Schematic representation of dynamic, reversible presentation of IKVAV using strand displacement. **b)** 3D Shadow projection of confocal sections of neurospheres grown on DNA-IKVAV platform (**ON**) before and after removing (**OFF**) the signal. $GFAP^+$ astroglial cells (red) and Tuj^+ neuronal cells (green) were reconstructed in 3D using the Imaris program (Bitplane scientific software). Nuclei were stained with DAPI (blue). **c)** Normalized area of neurospheres grown on substrates where the IKVAV signal is first removed (switched between **ON** and **OFF**) and then restored (switched back to **ON**). All values were normalized relative to the inner neurospheres in the same conditions. **d)** Immunofluorescence images of neurospheres on substrates where the IKVAV signal is dynamically switched from **ON** to **OFF** and back to **ON**. Laminin coatings were used as a positive control. (*** $p < 0.001$). Scale bars for fluorescent images: 50 µm, relevant to all images. Scale bars for brightfield images: 40 µm.

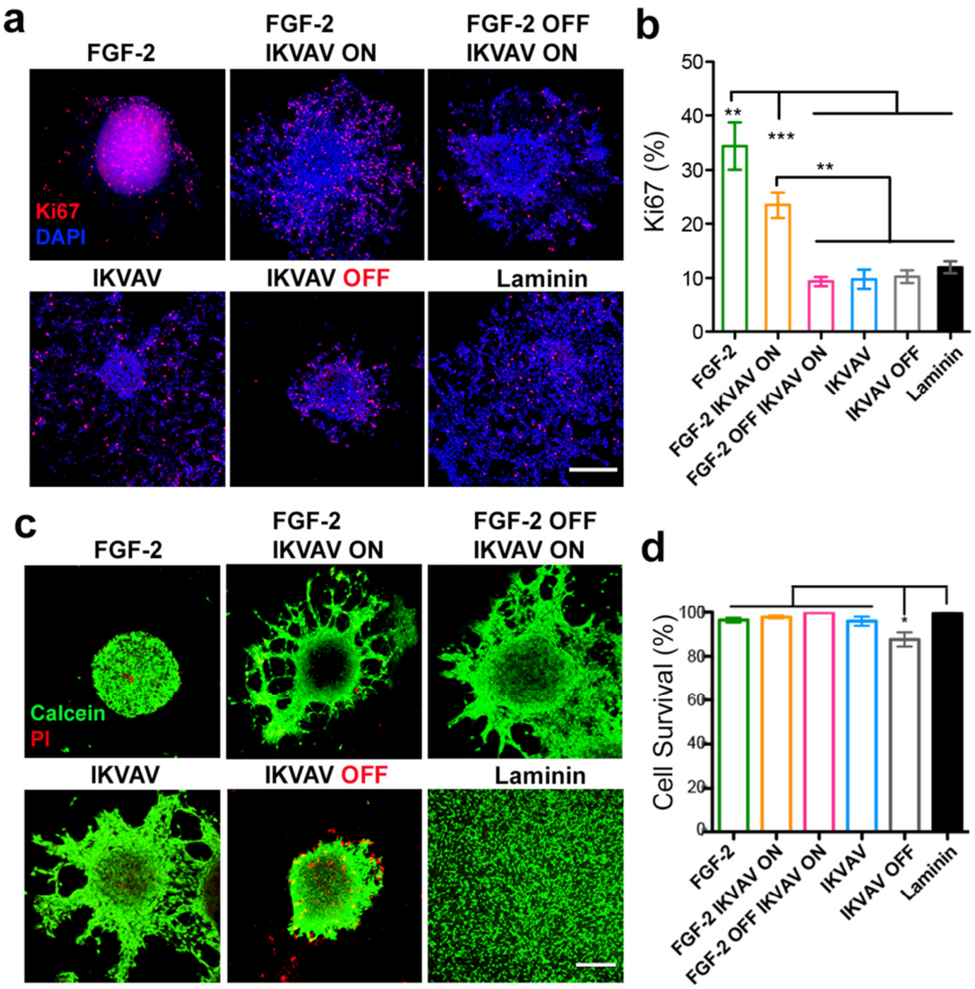

Supplementary Fig. 18: NSC survival and proliferation on P-DNA platform. Immunofluorescence images of neurospheres on a P-DNA platform presenting 1 or 2 signals sequentially or simultaneously stained with **a)** Ki67 marker (for proliferation) and DAPI (nuclear marker) and **b)** Calcein (live cells, green) and PI (dead cells red). **c)** Percentage of Ki67+ proliferative cells and **d)** Cell survival on indicated surfaces. (* $p < 0.05$, ** $p < 0.01$, *** $p < 0.001$). Plots in (b) and (d) also appear in main Figure 4 and are shown here again for clarity. All scale bars: 50 µm, and are relevant to all images.

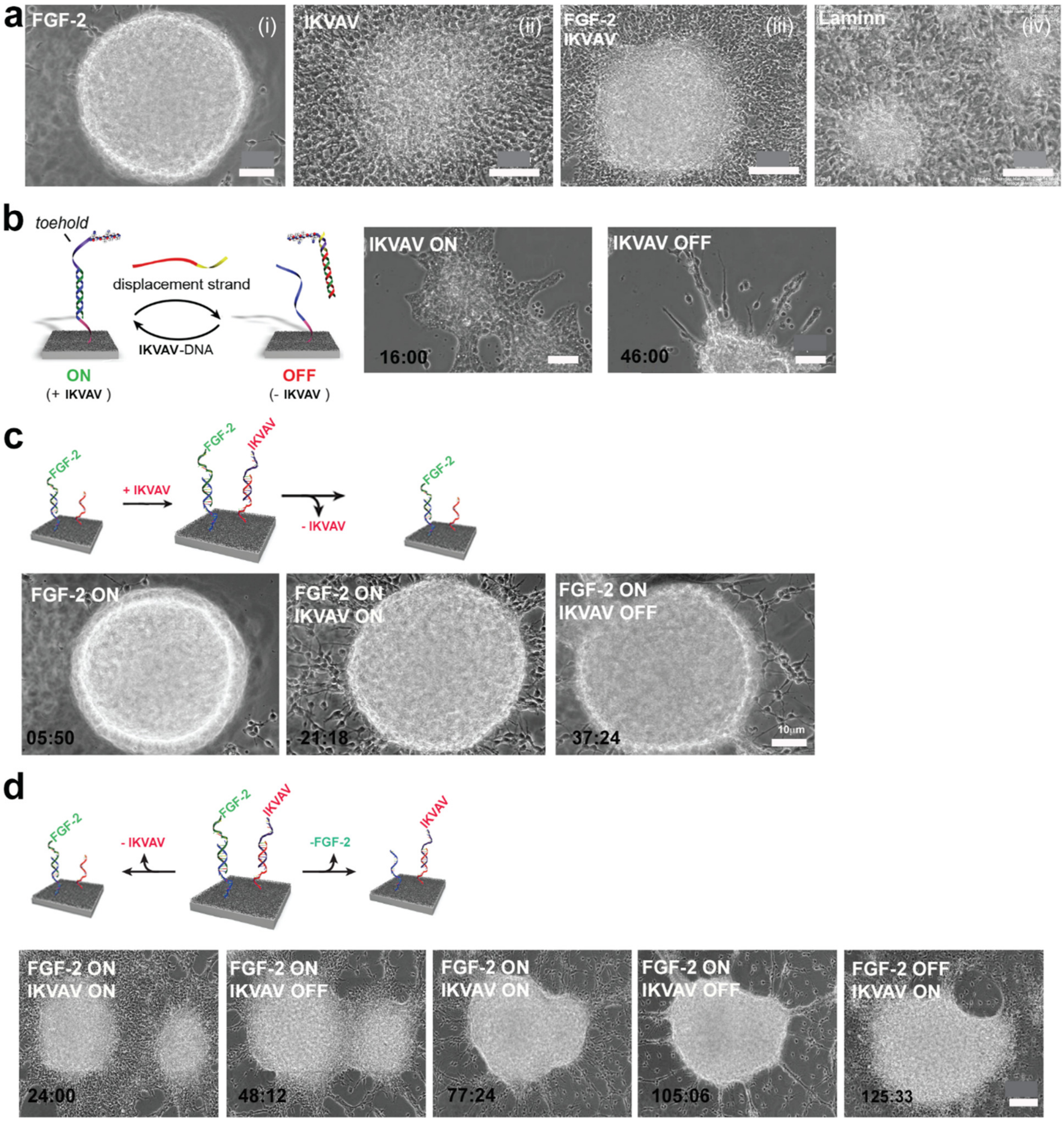

Supplementary Fig. 19: Bright-field, time-dependent analysis of neurosphere behavior on dynamic surfaces. a) Cell migration after 24hr on indicated surfaces. Scale bars: (i) 10 µm, (ii-iv) 40 µm. **b)** Cell migration at indicated time points with the IKVAV signal switched **ON** and then **OFF**. Scale bars: 40 µm. **c)** Cell migration out of and back into the neurosphere upon switching the IKVAV signal **ON** and then **OFF** in the presence of a constant FGF-2 signal. Scale bar: 10 µm, relevant to all images. **d)** Orthogonal, sequential control of IKVAV and FGF-2 signals at time points indicated. Scale bars: 30 µm, relevant to all images.

Strand name(s)	DNA sequence (5' to 3')	Components (Ex. Data Fig. 4)
DNAsurf1	GGCTGTCCACTGAGAGTCATGAC--[amine]	1A-1L
DNA1; X_DNA	CTCAGTGGACAGCCTTTTT--[amine]	1A*-1B*
DNA1-displ	AAAAAGGCTGTCCACTGAG	1B-1A
DNA1-FAM	CTCAGTGGACAGCCTTTTT--[FAM]	
DNAsurf2	GGCTGTCCACTGAG--[amine]	2L*
DNAhp	CTCAGTGGACAGCCGCGAGGTAAAACGACGGCCAGTCCTCGCTT--[amine]	2L-2A-2B-2A*
DNAhp-open	ATATATATGCGAGGACTGGCCGTCGTTTTA	2C*-2A-2B*
DNAhp-open-displ	TAAAACGACGGCCAGTCCTCGCATATATAT	2B-2A*-2C
DNAhp-FRET	[FAM]--CTCAGTGGACAGCCGCGAGGTAAAACGACGGCCAGTCCTCGCTT--[BQH]	
DNAsurf3	[amine]--AGTTTCGTCCAACGCTCCAGAACTTTTTT	3L-3A-3B
DNA3; Y_DNA	[amine]--TTTTTGTTCTGGAGCGTTG	3C-3A*
DNA3-displ	CAACGCACCAGAACAAAAA	3A-3C*
DNA3b-TMR	GTTCTGGAGCGTTGGACGAAACTTTTTT--[TMR]	
SYNERGY_0	CAGTACTGAAGTTTCGTCCAACGCTCCAGAACGGCTGTCCACTGAG	
SYNERGY_3	CAGTACTGAAGTTTCGTCCAACGCTCCAGAACTGAGGCTGTCCACTGAG	(variable region for *N* in green)
SYNERGY_11	CAGTACTGAAGTTTCGTCCAACGCTCCAGAACAGTACTGAGGCTGTCCACTGAG	
SYNERGY_15	CAGTACTGAAGTTTCGTCCAACGCTCCAGAACATTCAGCAGTACTGAGGCTGTCCACTGAG	
SYNERGY_21	CAGTACTGAAGTTTCGTCCAACGCTCCAGAACGGCTGTCCACTGAG	(same as SYNERGY_0)
SYNERGY_36	CAGTACTGAAGTTTCGTCCAACGCTCCAGAACATTCAGCAGTACTGAGGCTGTCCACTGAG	(same as SYNERGY_15)
DNA4a	[amine]--TTTTTGTTCTGGAGCGTTGGACGAAA	
DNA4b	GTTCTGGAGCGTTGGACGAAATTTTT--[amine]	
DNA4a-TMR	[TMR]--TTTTTGTTCTGGAGCGTTGGACGAAA	
DNA4b-TMR	GTTCTGGAGCGTTGGACGAAATTTTT--[TMR]	

Supplementary Table 1: Sequences of all DNA strands used. Names and corresponding sequences of all DNA strands used in this manuscript. Where relevant, the color coded components as described in Extended Figure 4 are shown. Amine modifications are indicated at the corresponding end of the DNA. Abbreviations: FAM = fluorescein; TMR = TAMRA; BHQ = Black Hole Quencher.

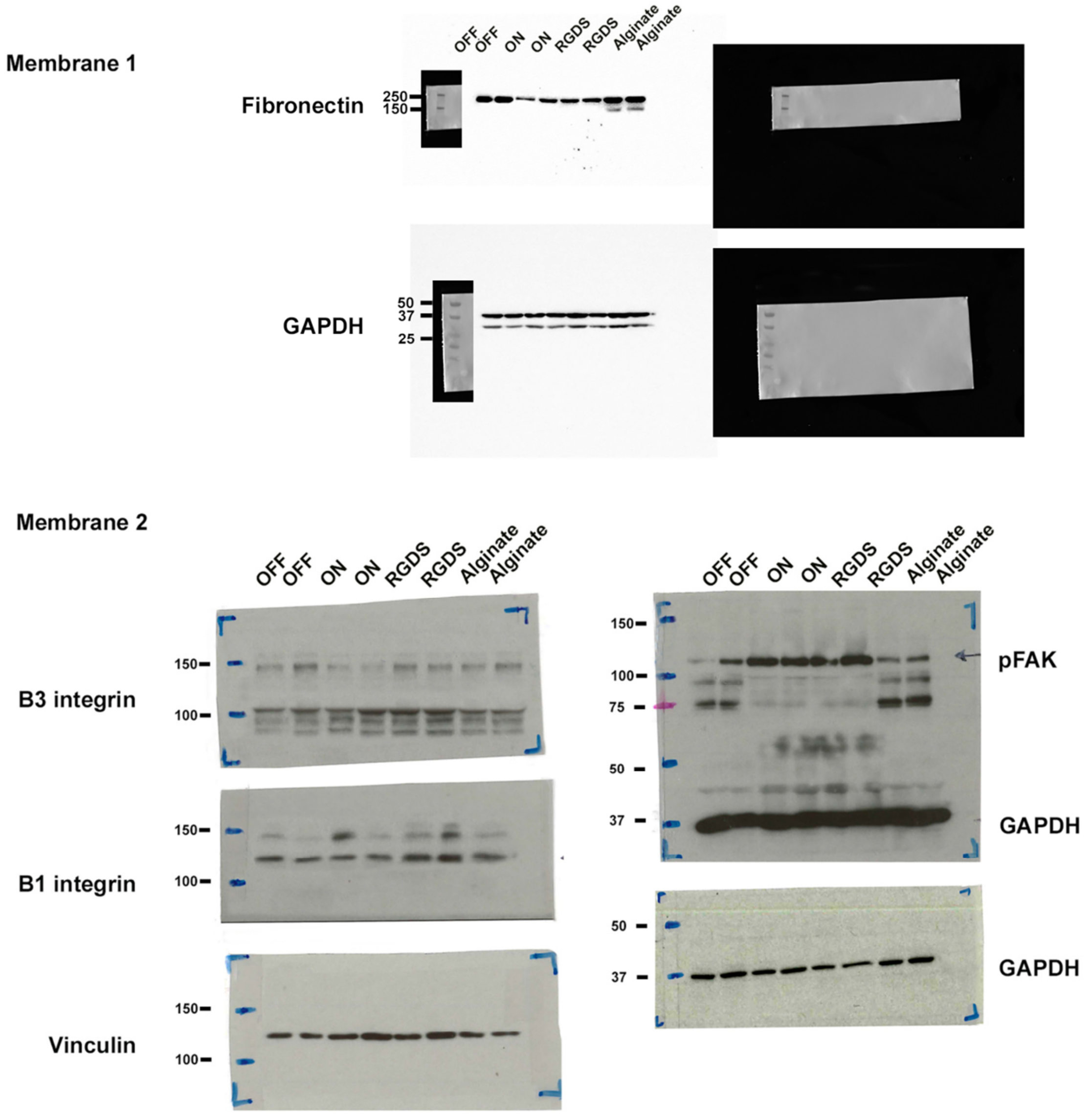

Entire blot from Main Figure 2g.

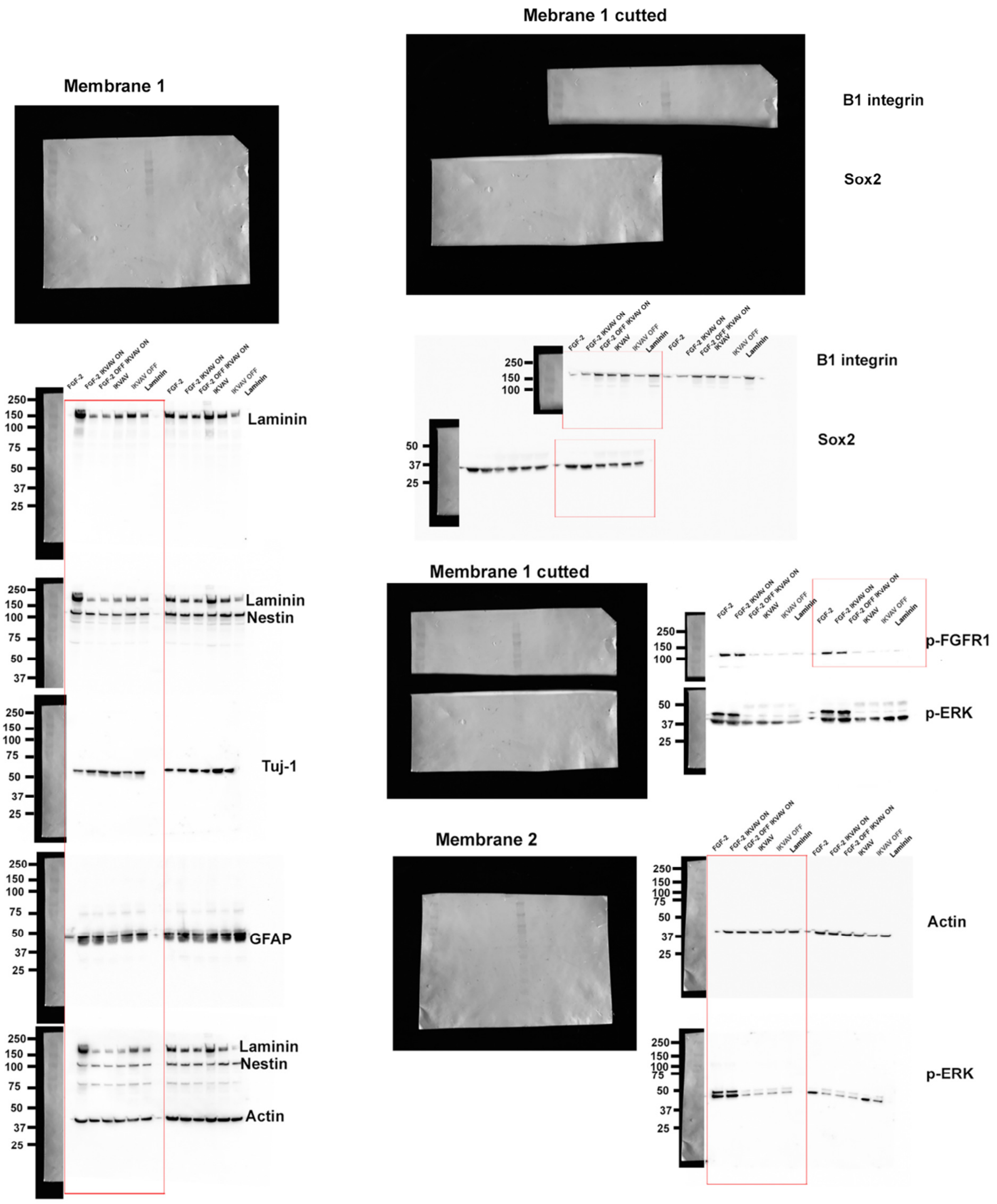

Entire blot from Main Figure 4i-j.
